# Oncogenic and microenvironmental signals drive cell type specific apoptosis resistance in juvenile myelomonocytic leukemia

**DOI:** 10.1038/s41419-025-07479-2

**Published:** 2025-03-08

**Authors:** Naile Koleci, Ying Wu, Niels Anton Wehner, Jovana Rajak, Venugopal Rao Mittapalli, Julia Mergner, Hui Xiao, Jun Wang, Madeleine Wahl, Sheila Bohler, Konrad Aumann, Georg Häcker, Senthilkumar Ramamoorthy, Melanie Boerries, Susanne Kirschnek, Miriam Erlacher

**Affiliations:** 1 Department of Pediatrics and Adolescent Medicine, Division of Pediatric Hematology and Oncology, University Medical Center Freiburg, Faculty of Medicine, University of Freiburg, Freiburg, Germany; 2 Faculty of Biology, University of Freiburg, Freiburg, Germany; 3 Bavarian Center for Biomolecular Mass Spectrometry, Technical University of Munich, Freising, Germany; 4 Department of Pathology, Institute for Clinical Pathology, University Medical Center Freiburg, Freiburg, Germany; 5 Institute for Medical Microbiology and Hygiene, University of Freiburg, Freiburg, Germany; 6 Institute of Medical Bioinformatics and Systems Medicine, Medical Center University of Freiburg, Faculty of Medicine, University of Freiburg, Freiburg, Germany; 7 German Cancer Consortium (DKTK) and German Cancer Research Center (DKFZ), Partner Site, Freiburg, Germany; 8 Department of Pediatrics and Adolescent Medicine, University Medical Center Ulm, Ulm, Germany; 9 Present Address: Department of Medicine III, Hematology and Oncology, TUM University Hospital, Technical University of Munich, TUM School of Medicine and Health, Munich, Germany; 10 Present Address: Center for Translational Cancer Research (TranslaTUM), Technical University of Munich, Munich, Germany

**Keywords:** Preclinical research, Cancer, Leukaemia

## Abstract

Juvenile myelomonocytic leukemia (JMML) is caused by constitutively activated RAS signaling and characterized by increased proliferation and predominant myelomonocytic differentiation of hematopoietic cells. Using *MxCre;Ptpn11*^*D61Y*/+^ mice, which model human JMML, we show that RAS pathway activation affects apoptosis signaling through cell type-dependent regulation of BCL-2 family members. Apoptosis resistance observed in monocytes and granulocytes was mediated by overexpression of the anti-apoptotic and down-regulation of the pro-apoptotic members of the BCL-2 family. Two anti-apoptotic proteins, BCL-X_L_ and MCL-1, were directly regulated by the oncogenic RAS signaling but, in addition, were influenced by microenvironmental signals. While BCL-X_L_ and BCL-2 were required for the survival of monocytes, MCL-1 was essential for neutrophils. Interestingly, stem and progenitor cells expressing the oncogenic PTPN11 mutant showed no increased apoptosis resistance. BCL-X_L_ inhibition was the most effective in killing myeloid cells in vitro but was insufficient to completely resolve myeloproliferation in vivo.

## Introduction

Juvenile myelomonocytic leukemia (JMML) is a rare and highly aggressive myeloid neoplasia of early childhood, characterized by constitutive active RAS signaling. Mutations in *KRAS*, *NRAS*, *RRAS*, or *RRAS2* or in their regulators *PTPN11* (encoding for SHP2), *CBL*, or *NF1* [1–7] in JMML cells, enhance proliferation and myelomonocytic differentiation upon cytokine stimulation (i.e., GM-CSF) [8]. Monocytes and granulocytes infiltrate bone marrow (BM), spleen, liver and other organs, causing fever, thrombocytopenia, hepatosplenomegaly, respiratory distress, and diarrhea [1, 9–12]. Most patients require allogeneic stem cell transplantation (HSCT).

Resistance to apoptosis is a key cancer hallmark contributing to tumor emergence and persistence [13–15]. The intrinsic apoptosis pathway is controlled by BCL-2 family proteins [16–21], divided into anti-apoptotic (BCL-2, BCL-XL, MCL-1), pro-apoptotic BH3-only (BIM, PUMA, BID, NOXA, BMF, BAD, BIK, HRK), and pro-apoptotic effector proteins (BAX, BAK, BOK) [22–29]. Once activated, effector proteins multimerize and lead to mitochondrial outer membrane permeabilization, cytochrome c release, and cell death [28, 30]. Pro- and anti-apoptotic proteins interact to regulate cell fate. Every cell requires one or more BCL-2 protein/s for survival. In cancer cells, this “BCL-2 protein addiction” is modulated by the driver mutations and microenvironmental signals [31–33]. The fact that cancer cells, at the same time, accumulate pro-apoptotic proteins (known as “mitochondrial priming”) is used during cancer therapy since cytotoxic drugs induce apoptosis more readily in cancer than in healthy cells.

RAS activation influences cell death decisions by regulating BCL-2 proteins, by inhibiting pro-apoptotic and activating anti-apoptotic proteins, as observed mostly in cell lines [34, 35]. Here, we investigated BCL-2-regulated apoptosis in a genetically engineered mouse model for JMML. Using mice expressing the PTPN11/SHP2^D61Y^ mutation in the hematopoietic system [1, 36], we found survival benefits in leukemic monocytes and neutrophils, mediated by higher BCL-X_L_ and/or MCL-1 expression. Adjacent non-leukemic cells acquired similar but transient apoptosis resistance. Surprisingly, SHP2^D61Y^ expressing hematopoietic stem and progenitor cells (HSPCs) showed no apoptosis resistance. Inhibition of BCL-2 proteins by selective BH3-mimetics revealed differential “addiction” in monocytes and neutrophils. In vivo treatment with the BCL-X_L_ inhibitor A1155463 reduced myeloproliferation but did not cure the mice. In conclusion, apoptosis resistance in JMML is restricted to few cell types and influenced by microenvironmental signals.

## Methods

### Genetically engineered JMML mouse model

Animal procedures were carried out in accordance with the regulatory requirements of German law. *MxCre* and *Ptpn11*^*D61Y/+*^ were provided by Benjamin Neel and bred to obtain *MxCre;Ptpn11*^*D61Y/+*^. All mice received three doses of poly I: C (300 μg/dose/mouse) every other day. Leukemic mice were euthanized and analyzed at different disease stages (Supplementary Fig. 6). All mice were euthanized when showing distress.

### Mice genotyping

The DNA from ear punches was PCR assayed for Neo-Stop cassette and *MxCre* using the primers shown in Supplementary Table 4 (Supplementary Fig. 6).

### In vivo treatment

The BCL-X_L_ inhibitor A1155463 (Selleck Chemicals) was dissolved in 2% DMSO, 30% PEG300, 2%Tween80 and administered intraperitoneally (ip), daily for 28 days (5 mg/kg/day). Mice were euthanized one day after treatment, and hematopoietic cell compartments were analyzed.

### Transplantation JMML mouse model

CD45.1+ (WT) mice were sub-lethally irradiated (3 Gy). Leukemic splenocytes from terminally ill S3-*MxCre;Ptpn11*^*D61Y/+*^ mice were isolated and transplanted intravenously (1 × 10^6^ CD45.2 in PBS) into the irradiated mice. Recipients received riketron in drinking water for four weeks after irradiation. Engraftment and myeloproliferation were assessed in peripheral blood via flow cytometry.

### Flow cytometry

For *surface staining,* single-cell suspensions from BM, spleen, blood, and liver and lung MNCs were stained with antibodies as in Supplementary Table 3, and the gating strategy was performed as shown on Supplementary Fig. 7. For *intracellular staining,* cells were stained for surface markers, then fixed and permeabilized using a buffer set (eBioscience concentrate and diluent), followed by intracellular protein staining per the manufacturer’s instructions. For *phospho-flow*, cells were stained for surface markers, incubated with 3.7% formaldehyde, then 90% cold methanol, and stained with pMAPK and pmTOR phosphoantibodies. For *apoptosis staining*, after surface staining, cells were resuspended in binding buffer (BB) containing Annexin V (AV) and 7AAD, incubated for 15 min, and measured on a BD Fortessa. Freshly isolated CD11b^+^ or LSK cells were cultured for the specified time points before apoptosis staining.

### Cell isolation and culture

#### LSK cells

Lineage-negative (Lin^-^) cells were isolated from BM using the lineage depletion Kit (Miltenyi Biotec). Followed by sorting of Sca^+^cKit^+^ cells and cultured in IMDM medium (Gibco), with 10% FCS (Thermo Fisher), 1% penicillin/streptomycin (ThermoFisher), stem cell factor (SCF), thrombopoietin (TPO) and Fms-related receptor tyrosine kinase 3 ligand (FLT3L) at 100 ng/ml each (ImmunoTools).

#### CD11b^+^ cells

CD11b^+^ myeloid cells were isolated using a MACS kit (Miltenyi Biotec) with two column separation rounds and cultured in IMDM medium supplemented with 30% FCS, 1% P/S, 25 mM HEPES (Sigma-Aldrich), 0,1 mM MEM non-essential amino acids (Gibco) and 0,1 mM sodium pyruvate (Gibco), 2-B-mercaptoethanol (Gibco). Where indicated, CD11b^+^ cells were stained for CD45.1 and CD45.2 and sorted. CD11b^+^ CD45.1 and CD45.2 were cultured individually or in co-cultures. Neutralizing antibodies for anti-CD74 (R&D systems), anti-ITGAM (Invitrogen) or TNFα (Immunotools) were added as indicated.

### Apoptosis induction

Apoptosis of LSK and CD11b^+^ cells was assessed after treatment with BH3-mimetics: ABT737 (Selleck Chemicals), ABT199 (Selleck Chemicals), A115546, S63845 (Synthesis) and Runx inhibitor (Tocris). After BH3 mimetic treatment, cells were stained for myeloid markers, stained with AV and 7AAD, and measured on a flow cytometer. Specific apoptosis was calculated as: 100 × (% live cells without treatment − % live cells with treatment)/% live cells without treatment.

### Cytokine bead array

Cytokines found in BM and spleen secretomes were quantified using a cytokine bead array (CBA) kit, according to the manufacturer’s instructions (BD Bioscience).

### RT-MLPA

mRNA was isolated from BM-LSK cells and spleen CD11b^+^ of *MxCre* and S3 *MxCre;Ptpn11*^*D61Y/+*^ mice using Fast Spin columns (Zymo Research) and reverse transcribed into cDNA for RT-MLPA (MRC-Holland). cDNA was ligated to two oligonucleotides, and the amplicons were separated by capillary electrophoresis (ABI-3130xl Genetic Analyzer). Data was analyzed using Sequence Pilot software (JSI Medical Systems) and normalized taking the sum of all peaks as 100%.

### Western blot

CD11b^+^ myeloid cells were lysed with 1x Laemmeli buffer and sonicated. Lysates were resolved in 12% SDS-PAGE and transferred to PVDF membrane, which were incubated overnight with primary antibodies diluted in 5% BSA in TBST: Bim (C.Signaling Technology), Bid (BD), Noxa (Enzo), Bmf (NBPI), Bak and Bax (C.Signaling Technology). Mouse or rabbit HRP-conjugated secondary antibodies were incubated with the respective membranes. β-actin (Sigma-Aldrich) was used as a loading control. Membranes were developed using Fusion equipment. For protein determination, equal volumes extracted from CD11b^+^ cells were run per Western blot, and β-actin band intensities were quantified using ImageJ. Supplementary Table 2 lists the antibodies.

### RNA isolation and sequencing

#### RNA isolation

RNA was isolated from CD11b^+^ cells (BM and spleen of S3 *MxCre;Ptpn11*^*D61Y/+*^ and *MxCre* mice; 3 mice/genotype) with the RNeasy Micro kit (Qiagen) following the kit instructions. RNA sequencing was performed at the Genomic and Proteomic Core Facility of the German Cancer Research Center.

### RNA Sequencing and data analysis

RNA sequencing libraries were prepared using the TruSeq Stranded mRNA Library Prep Kit (Illumina) according to the manufacturer’s protocol. The 100 bp paired-end reads were trimmed using TrimGalore (v0.6.5) and mapped to the mouse reference genome (GRCm38) using STAR (v2.5.3a). Read counts were normalized, and differential expression was calculated using the R packages DESeq2 (v1.22.2) and limma (v3.38.3). Gene set enrichment analysis was performed using GAGE tools (v2.44.0). The next-generation sequencing data is deposited at NCBI GEO (GSE277667).

### Whole exome sequencing (WES) and data analysis

Genomic DNA was extracted from splenocytes of control *MxCre* and *MxCre;Ptpn11*^*D61Y/+*^ S3 mice by using the DNeasy Blood &Tissue Kit (Qiagen). Skin DNA was used as a germline control. WES libraries were prepared using the Agilent SureSelectXT Mouse All Exon kit. Adapter and quality trimming were performed using Trimmomatic (v0.39), and the trimmed reads were mapped to the mouse reference genome (GRCm38) using the BWA-MEM aligner. The aligned reads were processed using Samtools and the GATK toolkit (v3.8.1). Variants were identified using Samtools mpileup and VarScan (v2.4.3) and annotated with Annovar and SnpEff (v4.3).

### BM and spleen secretome

Two femurs per mouse were flushed with 2 ml PBS to collect secretome content. Spleens were smashed and resuspended in 2 ml PBS. The supernatants were used for TNFα cytokine assay or further processed for mass spectrometry.

### Mass spectrometry-based proteomics

Tryptic peptides from secretome lysate were labeled with TMT10plex reagent, pooled and fractioned as described previously [74] [75]. Nanoflow LC-MS/MS was performed on a Dionex 3000 (Thermo) coupled online to an Orbitrap Fusion LUMOS (Thermo). Samples were measured in DDA mode with a MS3 method and 50 min linear gradient. Database search against the mouse reference proteome (UP000000589) and common contaminants was performed with MaxQuant (v. 1.6.3.3) with standard settings for reporter ion MS3 [78]. The spleen and bone marrow TMT set corrected TMT reporter intensities were total sum and row-wise normalized for statistical analysis. The mass spectrometry proteomics data have been deposited to the ProteomeXchange Consortium via the PRIDE (PMID 34723319) partner repository with the dataset identifier PXD055845.

### Histology

BM/sternum, spleen, liver, and lung sections from indicated mice were fixed in 4% paraformaldehyde. The samples were decalcified in EDTA for 5–10 days and embedded in paraffin. The sections were stained with haematoxylin and eosin (H&E). Images were acquired using a Zeiss microscope.

### Statistics

The unpaired/non-parametric Mann-Whitney test was used to analyze all experiments. *P* values: *P < 0.05, **P < 0.01, ***P < 0.001, ****P < 0.0001. Analysis was performed on GraphPad Prism.

## Results

### Monocytes and granulocytes isolated from *MxCre;Ptpn11*^*D61Y/+*^ mice show increased apoptosis resistance

**Fig. 1:**
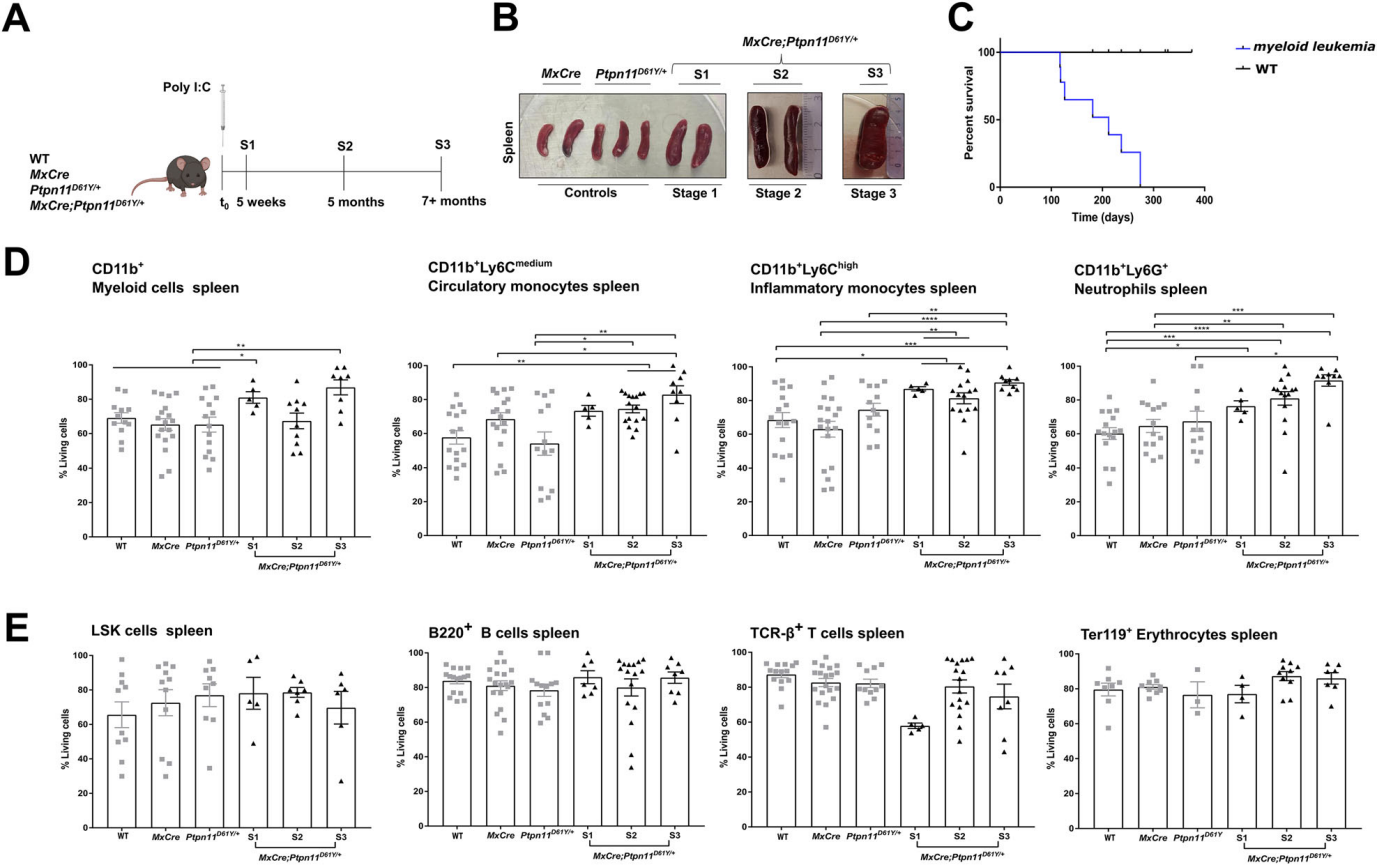
Higher viability rates are found in splenic myeloid cells from SHP2^D61Y^ mice compared to controls. **A** Schematic representation of the experimental procedure. All experimental mice (controls -WT, *MxCre*, *Ptpn11*^*D61Y/+*^ and JMML mice - *MxCre;Ptpn11*^*D61Y/+*^) were injected with 300 μg poly I:C/mouse (in 3 doses every other day) to activate the oncogenic mutation. The experimental mice were analyzed at different stages of the disease: preleukemic - defined as stage 1 - S1 (5 weeks after polyI:C injection); leukemic - defined as stage 2 - S2 (5 months after polyI:C injection) and full-blown myeloproliferative disease - defined as stage 3 - S3 (7+ months after poly I:C injection - terminally ill mice). **B** Representative spleens are shown for: control mice (*MxCre, Ptpn11*^*D61Y/+*^) and JMML mice *MxCre;Ptpn11*^*D61Y/+*^ (S1, S2, S3). **C** Survival curves are shown for control mice (WT) and JMML *MxCre;Ptpn11*^*D61Y/+*^ mice. The percentage of live cells (AV^−^ 7AAD^−^) was determined in the following splenic hematopoietic cell types: **D** CD11b^+^ myeloid cells; CD11b^+^Ly6C^medium^ - circulatory monocytes; CD11b^+^Ly6C^high^ - inflammatory monocytes and CD11b^+^Ly6G^+^ - neutrophils; **E** LSK - stem and progenitor hematopoietic cells, B220^+^ - B cells, TCR-β^+^ - T cells and Ter119^+^ - erythrocytes obtained from the spleen. All the above cell populations were determined ex vivo and assayed by flow cytometry for the indicated genotypes. The gating strategy was performed as described in Supplementary Fig. 7. All data are presented as mean ± SEM (*n* = 3–10; ≥5 independent experiments).

Mice expressing SHP2^D61Y^ in the hematopoietic system and known to develop JMML-like myeloproliferation were generated by treating *MxCre;Ptpn11*^*D61Y/+*^ mice with PolyI:C [1]. We defined terminally ill mice as “stage 3” (S3) and earlier disease stages as “stage 1 and 2” (S1, S2), respectively (Fig. 1A). *MxCre;Ptpn11*^*D61Y/+*^ mice developed massive splenomegaly and succumbed 130–400 days post polyI:C injection (median 274 days, Fig. 1B, C). Flow cytometry revealed increasing infiltration of monocytic, granulocytic, and erythroid progenitors in the spleen, lung, and liver (Supplementary Fig. 1A–F). The number of HSPCs defined as lineage marker negative, Sca1 and cKit positive (LSK) cells, remained stable in the BM but increased in the S2–3 spleens (Supplementary Fig. 1G). Activated SHP2 signaling was confirmed by increased MEK and mTOR phosphorylation in SHP2^D61Y^ expressing myeloid cells (Supplementary Fig. 1H). Histopathology confirmed myelomonocytic organ infiltration (Supplementary Fig. 2). Flow cytometry immediately after organ isolation revealed higher viability of monocytes and neutrophils from spleen, especially in S2-3 mice (Fig. 1D). BM myeloid cells were generally more viable, with no significant differences between the genotypes (Supplementary Fig. 3A). SHP2^D61Y^ expressing LSK cells, lymphocytes and erythroid progenitors from the spleen (Fig. 1E) and BM (Supplementary Fig. 3B) showed no survival benefit.

**Fig. 2:**
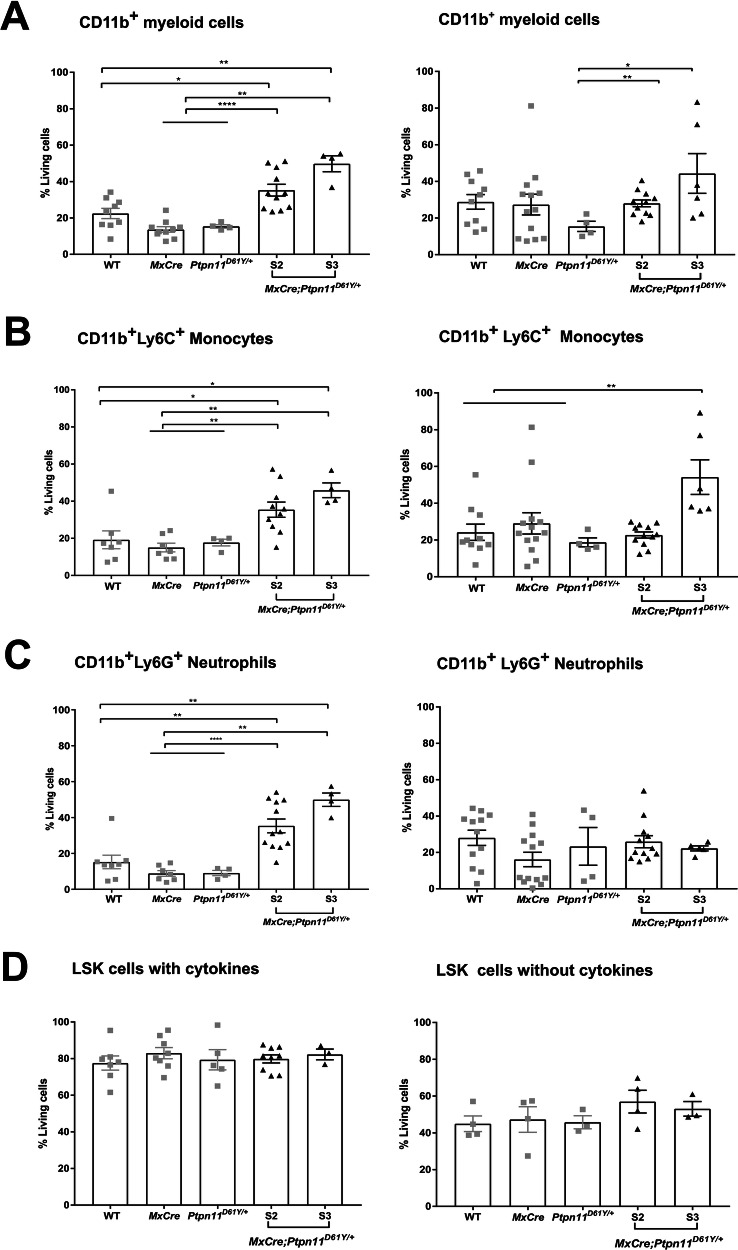
Higher survival rates are found in cultured primary murine myeloid cells expressing SHP2^D61Y^. The primary murine LSK and CD11b^+^ cells were cultured for 24 h and 48 h. The percentage of living cells (AV^-^7AAD^-^) was determined by flow cytometry for the following cell populations: **A** myeloid cells - CD11b^+^ cells; **B** monocytes - CD11b^+^Ly6C^+^; **C** neutrophils - CD11b^+^Ly6G^+^ after for 24 h (left panel) and 48 h (right panel) of culture**. D** LSK-stem and progenitor cells were cultured for 48 h in the presence and absence of cytokines (FLT3L, TPO and SCF), and living cells were determined as AV^-^7AAD^-^ by flow cytometry. The gating strategy was performed as described in Supplementary Fig. 7. All data are presented as mean ±SEM (*n* = 1–10; ≥3 independent experiments).

To confirm their reduced susceptibility to apoptosis, myeloid cells from S2 and S3 spleens were cultured for 24 and 48 h without pro-survival cytokines. Increased survival of S2-3 bulk myeloid cells (CD11b^+^), monocytes and granulocytes were observed compared to controls (Fig. 2A–C), with stronger effects in S3 cells. SHP2 mutant LSK cells showed no viability differences when cultured with or without cytokines compared to controls (Fig. 2D). Clonal evolution as the reason for the increased apoptosis resistance in late disease stages was excluded by whole exome sequencing (WES). In splenocytes of three S3 mice, no JMML-associated mutations (i.e., secondary RAS pathway lesions, mutations in *JAK2*, *JAK3*, or *SETBP1*) [37] were identified. Mutation numbers and variant allele frequencies (VAF) were similar in leukemic and control mice (Supplementary Fig. 3C, D, Supplementary Table 1).

### BCL-X_L_ and MCL1 are upregulated in SHP2^D61Y^ expressing myeloid cells

**Fig. 3:**
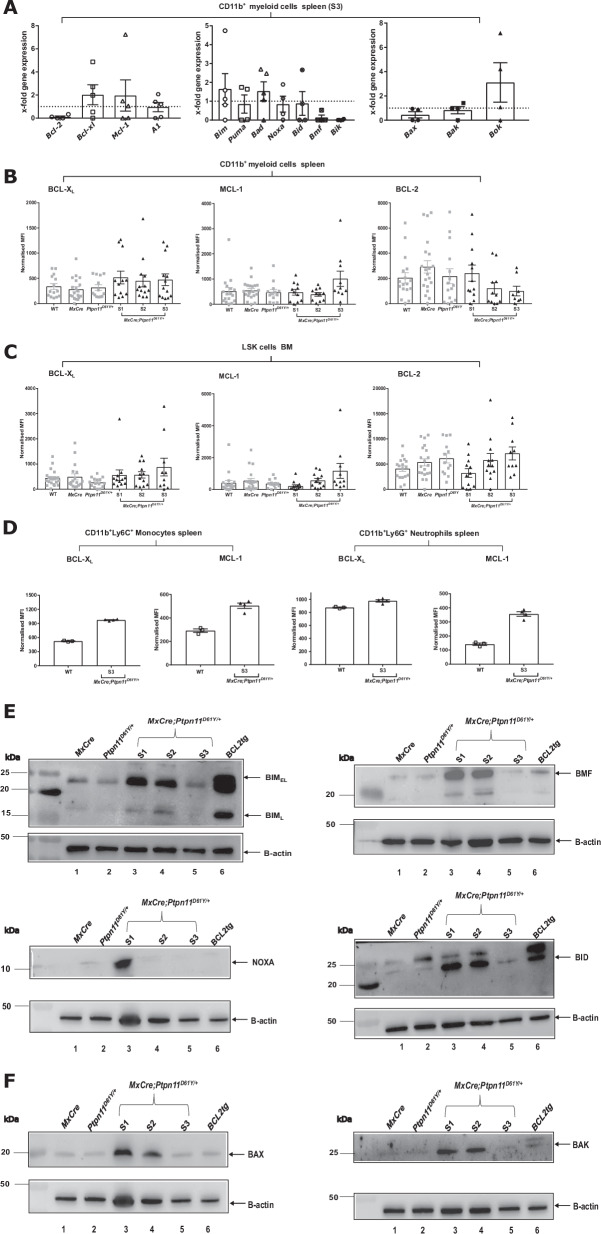
BCL-2 family proteins are deregulated in primary murine myeloid cells expressing SHP2^D61Y^. **A** The mRNA levels of BCL-2 family members (anti-apoptotic, pro-apoptotic and pore-forming) were determined by RT-MLPA in primary CD11b^+^ myeloid murine cells. Myeloid cells were isolated from S3 *MxCre;Ptpn11*^*D61Y/+*^ and control *MxCre* mice. Protein levels of pro-survival BCL-2 family proteins (BCL-X_L_, MCL-1 and BCL-2) were determined ex vivo by intracellular flow cytometry for the following cell types: **B** CD11b^+^ myeloid cells and **C** LSK–stem and progenitor cells. **D** The freshly isolated murine CD11b^+^ cells from disease stage 3 were cultured for 24 h and the pro-survival BCL-2 family proteins (BCL-X_L_, MCL-1) were determined in CD11b^+^Ly6C^+^ - monocytes and CD11b^+^Ly6G^+^ - neutrophils. Representative western blots showing the expression levels of pro-apoptotic BCL-2 proteins for the following BCL-2 family members: **E** BIM_EL_ and BIM_L_, BMF, NOXA, BID and pore-former proteins **F** BAX and BAK. BCL2 tg cells are the positive control for western blot. All data are presented as Mean ±SEM (*n* = 1–10; ≥3 independent experiments). The western blot analysis were performed on freshly isolated primary murine CD11b^+^ cells for an *n* = 2 with 5–7 mice/lysates. Antibodies used for WB are listed in Supplementary Table 2.

To understand SHP2^D61Y^ mediated apoptosis resistance at the molecular level, we analyzed levels of pro- and anti-apoptotic BCL-2 proteins. RT-MLPA revealed an approximately twofold increase of *Bcl-xl* and *Mcl-1* mRNA in spleen-isolated CD11b^+^ cells. While proapoptotic antagonists *Bim*, *Bad* and *Bok* were mildly but not significantly upregulated (Fig. 3A). Increased mRNA levels of *Bcl-2*, *Bcl-xl*, *Mcl-1* and *A1*, but also *Bid* and *Bax* were observed in the non-resistant LSK cells (Supplementary Fig. 3E). BCL-X_L_ and MCL-1 protein levels were slightly but not significantly higher in CD11b^+^ and LSK cells from S1-S3 mice, when compared to controls (Fig. 3B, C). In cultured SHP2^D61Y^ monocytes, BCL-X_L_ and MCL-1 levels were higher than in controls. In neutrophils, only MCL-1 was upregulated (Fig. 3D). Western blot analysis revealed upregulation of several pro-apoptotic proteins in S1 myeloid cells but downregulation during disease progression (Fig. 3E, F).

### Apoptosis resistance is driven by the oncogenic signaling

**Fig. 4:**
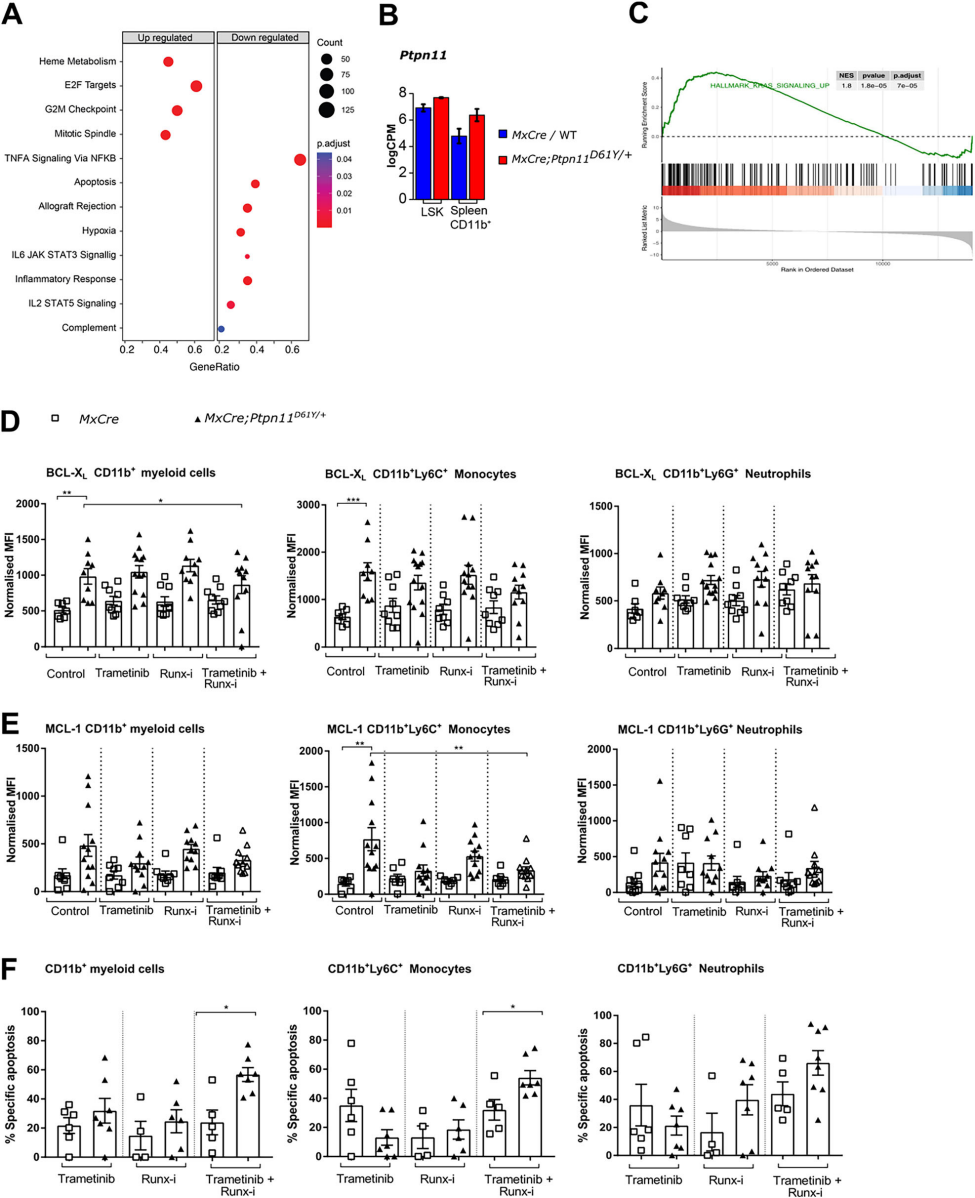
Combined treatment with MEK and RUNX1 inhibitors reduced BCL-X_L_ and MCL-1 expression in CD11b^+^ myeloid cells. **A** The bubble plot representing the top up-(left) and downregulated (top) hallmark gene sets in the comparison of S3 CD11b^+^ leukemic cells with the control (*Mxcre* or WT) cells. The gene ratio is represented on the y-axis, the bubble size corresponds to the gene count and the color map represents the adjusted *p* value. **B** The *Ptpn11* mRNA expression level in the bone marrow LSK and spleen CD11b^+^ cells in the S3 leukemic (*MxCre;Ptpn11*^*D61Y/+*^*)* and control (*MxCre or* WT) cells. The y-axis represents the logCPM (CPM- counts per million reads). **C** The gene set enrichment analysis of hallmark KRAS signaling up-regulated gene sets in the comparison of S3 spleen CD11b^+^ leukemic cells and LSK leukemic cells. The genes are ranked based on the fold change and the y-axis represents the running enrichment score (top) and the ranked list metric (bottom). The normalized enrichment score (NES), *p* value and adjusted p-value are shown in the table. Myeloid CD11b^+^ cells isolated from leukemic S2 mice or *MxCre controls* were treated with trametinib and/or Runx inhibitor for 24 h and then analyzed for expression of pro-survival proteins: **D** BCL-X_L_ and **E** MCL-1 and **F** % of specific apoptosis for myeloid cells (CD11b^+^ cells), monocytes (CD11b^+^Ly6C^+^) and neutrophils (CD11b^+^Ly6G^+^). All data are presented as Mean ±SEM (*n* = 1–10; ≥3 independent experiments).

RNAseq with gene set enrichment analysis (GSEA) revealed strong enrichment of cell cycle-related gene sets and downregulation of the apoptosis gene set in S3 leukemic spleen myeloid cells when compared to *MxCre-tg* controls (Fig. 4A). Unexpectedly, the levels of PTPN11 mRNA were higher in BM LSK than in splenic myeloid cells, this not correlating with the increased apoptosis resistance (Fig. 4B). Upregulated KRAS signaling (HALLMARK_KRAS_SIGNALING_UP) in myeloid cells versus immature LSK cells suggests that RAS/MAPK activity downstream of SHP2 explains their differing apoptosis susceptibility (Fig. 4C, Supplementary Fig. 4). To confirm that SHP2 and the RAS/MAPK signaling directly regulate BCL-X_L_ and MCL-1 expression, we inhibited MEK and, additionally the transcription factor RUNX1, which has been shown to drive myeloproliferation downstream of SHP2 [38]. BCL-X_L_ expression was reduced in bulk CD11b+ cells and specifically in monocytes, when both MEK and RUNX1 were inhibited (Fig. 4D). In contrast, MCL-1 expression was most profoundly reduced by MEK inhibition alone (Fig. 4E). Only combined MEK and RUNX1 inhibition induced apoptosis in both cell types (Fig. 4F).

### Microenvironmental signals contribute to apoptosis resistance in vivo

**Fig. 5:**
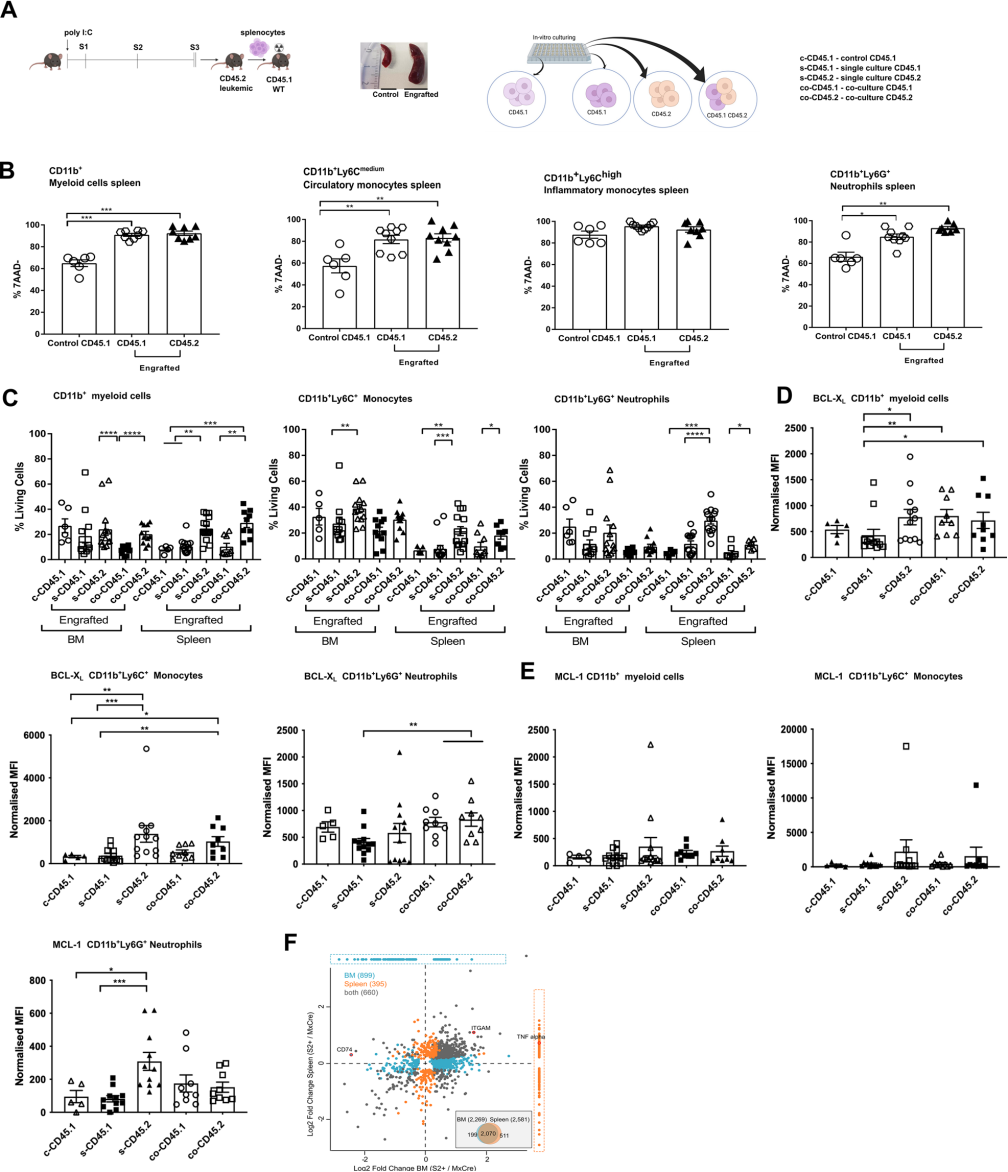
Cell-intrinsic and extrinsic signals drive the survival advantages of myeloid cells. **A** Schematic representation of the experimental procedure. *S*ub-lethally irradiated (3 Gy) WT (CD45.1) mice were transplanted with splenocytes (1 × 10^6^ cells) from S3 - terminally ill *MxCre;Ptpn11*^*D61Y/+*^ mice (CD45.2) (left panel). Representative spleens are shown for the control and the engrafted mice (middle panel). Schematic representation of the 24 h single cultures and co-cultures of leukemic - CD45.2 and WT - CD45.1 myeloid CD11b^+^ cells (right panel). The single cultures consisted of control CD45.1 - designated as c-CD45.1; single culture CD45.1- designated as s-CD45.1; single culture CD45.2 - designated as s-CD45.2. The co-culture conditions consisted of co-culture of CD45.1 and CD45.2 - designated as co-CD45.1 or co-CD45.2. The cells used for the condition: single cultures CD45.1 and CD45.2 were sorted from successfully engrafted recipient mice, while the control CD45.1 was obtained from not transplanted mice. Leukemic CD45.2 and WT CD45.1 myeloid cells were determined via flow cytometry. **B** Percentages of live cells (7AAD^-^) within the spleen residing CD45.1 and CD45.2 determined in engrafted and control mice for CD11b^+^ - myeloid cells, CD11b^+^Ly6C^medium^ - circulatory monocytes, CD11b^+^Ly6C^high^ - inflammatory monocytes, CD11b^+^Ly6G^+^ - neutrophils, was assessed ex vivo. **C** The CD11b^+^ cells were cultured for 24 h in single cultures or co-cultures. The percentage of living cells (AV^−^7AAD^−^) was determined by flow cytometry for: CD11b^+^ - myeloid cells; CD11b^+^Ly6C^+^ - monocytes and CD11b^+^Ly6G^+^ - neutrophils from BM and spleen. In vitro expression of pro-survival proteins: **D** BCL-X_L_ and **E** MCL-1 was determined in the myeloid compartment isolated from spleen, CD11b^+^ - myeloid cells, CD11b^+^Ly6C^+^ - monocytes and CD11b^+^Ly6G^+^ - neutrophils via intracellular staining after 24 h culture. **F** Significantly differently expressed proteins identified by mass spectrometry were selected and plotted based on log2 fold change obtained for BM or spleen supernatant from S3 leukemic vs MxCre. Significant changes in BM (turquoise), spleen (orange), or both (gray). Significant proteins only identified in either BM or spleen are marked by dashed boxes. The total number of identified proteins for BM and spleen are depicted as box insert. The gating strategy was performed as described in Supplementary Fig. 7. All data are presented as Mean ± SEM (*n* = 7–9; ≥3 independent experiments).

The generally higher viability of myeloid cells in BM than in the spleen prompted us to study the microenvironmental contribution to cell death decisions in leukemia. First, we transplanted leukemic mice into CD45.1 + WT recipients to generate chimeras (Fig. 5A, left). Suppl Fig. 5A shows the proportion of leukemic cells. Again, BM cells were more viable than spleen cells (Supplementary Fig. 5B). In chimeric spleens, increased survival rates were noted for both leukemic and recipient WT cells (Fig. 5B). Next, we performed co-culture experiments with WT and leukemic cells (Fig. 5A, right). We observed survival advantages (Fig. 5C) and elevated BCL-X_L_ and MCL-1 levels in leukemic myeloid cells, both in the absence and presence of WT cells (Fig. 5D, E). Interestingly, also WT cells and, particularly, neutrophils showed elevated BCL-X_L_ levels comparable to the ones of leukemic cells (Fig. 5D). Yet, they were not apoptosis-resistant (Fig. 5C), indicating that SHP2^D61Y^ expressing myeloid cells influence the survival of adjacent WT cells only in vivo. To identify pro-survival molecules in the leukemic microenvironment, we performed secretome mass spectrometry. Spleen supernatants contained more proteins than the BM supernatant (Fig. 5F). We identified proteins with known anti-apoptotic function at high abundance in leukemic spleens (i.e.ITGAM/CD11b, TNFα and CD74) and confirmed these findings by flow cytometry and cytokine assay (Fig. 5G, Supplementary Fig. 5C–F). Blocking either ITGAM or CD74 or adding TNFα in vitro did not induce apoptosis in WT or S3 myeloid cells (Supplementary Fig. 5G–I), indicating that other not yet identified microenvironmental signals affect cell survival within the leukemic spleen.

### BCL-X_L_ inhibition has potent anti-leukemic activity in vivo

**Fig. 6:**
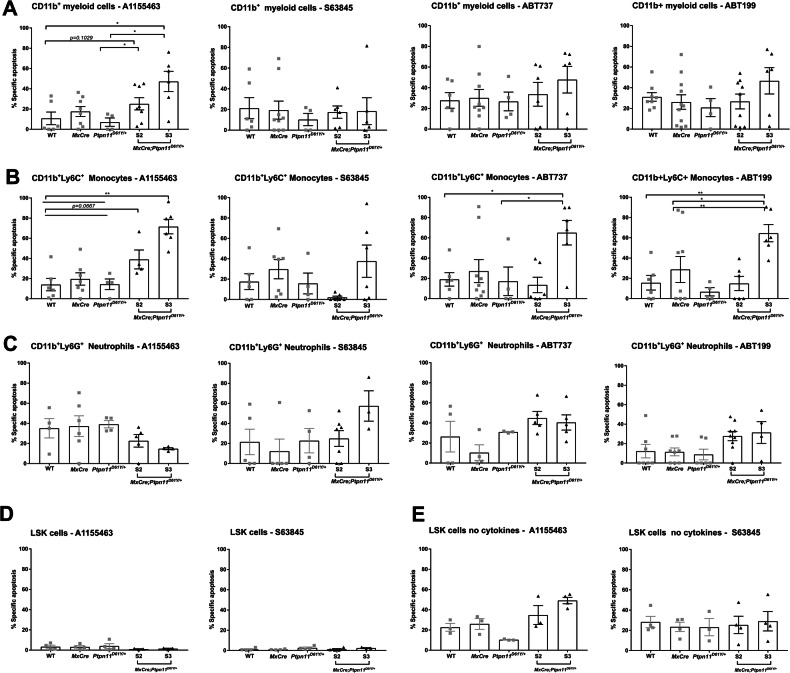
BH3 mimetics effectively kill myeloid SHP2^D61Y^ expressing cells in vitro. CD11b^+^ myeloid cells were treated with BH-3 mimetics, A1155463 (10 μM), S63845 (1 μM), ABT737 (3 μM), ABT199 (5 μM) for 48 h. Then, cells were stained for the surface markers CD11b^+^, Ly6G^+^, and Ly6C^+^ followed by staining for AV and 7AAD, and the viable cells were determined as AV^-^7AAD^-^ on flow cytometry. The percentage of specific apoptosis induced by BH3 mimetics was calculated for the specific cell types: **A** CD11b^+^ - myeloid cells, **B** CD11b^+^Ly6C^+^ - monocytes and **C** CD11b^+^Ly6G^+^ - neutrophils. Freshly isolated LSK stem and progenitor cells (cultured with or without cytokines) were treated with the BCL-X_L_ (A1155463) or MCL-1 (S63845) inhibitor for 48 h. The percentage of specific apoptosis was calculated for LSK cells: **D** in the presence (of TPO, FLT3, and SCF) and **E** in the absence of cytokines. The gating strategy was performed as described in Supplementary Fig. 7. All data are presented as Mean ±SEM (*n* = 1–3; ≥3 independent experiments).

**Fig. 7:**
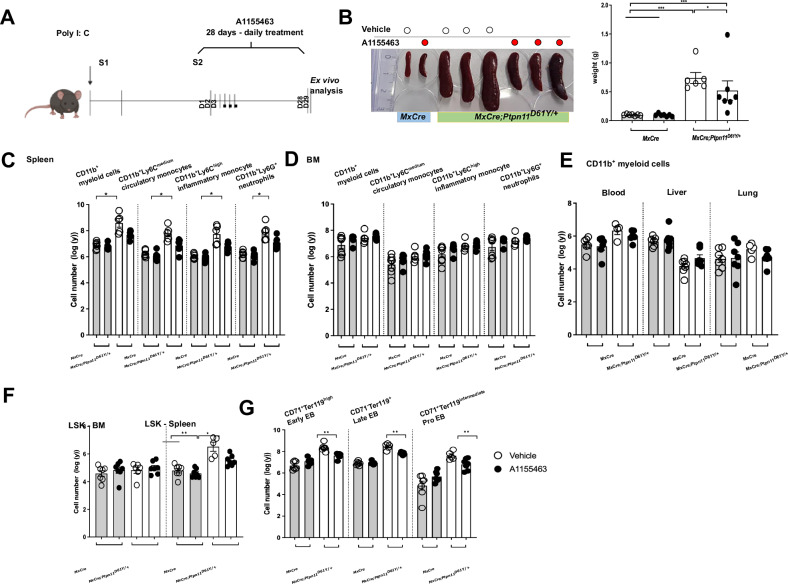
BCL-X_L_ inhibitor A1155463 significantly reduces myeloproliferation in JMML mice. **A** Schematic representation of experimental procedure. Mice were treated with the BCL-X_L_ inhibitor A1155463 (5 mg/kg/day) for 28 days (daily); on day 29 the mice were sacrificed and analyzed, and the leukemic burden was assessed. **B** Representative spleens (figure and weight) after in vivo treatment with the A1155463 or vehicle mice. Absolute cell counts are shown for the following cell populations of the BCL-X_L_ inhibitor A1155463 or vehicle-treated mice: myeloid cells - CD11b^+^ - myeloid cells; CD11b^+^Ly6C^medium^ – circulatory monocytes; CD11b^+^Ly6C^high^ – inflammatory monocytes; CD11b^+^Ly6G^+^ – neutrophils residing in the **C** spleen and **D** BM. **E** Number of CD11b+ myeloid cells residing in the blood, liver and lung. **F** LSK stem and progenitor hematopoietic cells. **G** Erythrocytes: - CD71^+^Ter119^high^ - Early erythroblasts (EB); (EB) - CD71^+^Ter119^intermediate^ - Pro-erythroblasts; CD71^-^Ter119^+^ - late EB. All data are presented as Mean ±SEM (*n* = 3–7; =3 independent experiments).

To identify the anti-apoptotic BCL-2 protein(s) required for leukemic cell survival, we used selective BH3-mimetics in vitro [39–44]. Splenic myeloid cells were most sensitive to BCL-X_L_ inhibition (Fig. 6A). Leukemic monocytes were sensitive to both combined and selective inhibition of BCL-X_L_ and BCL-2 (Fig. 6B), while neutrophils were most sensitive to MCL-1 inhibition (Fig. 6C). Greatest sensitivity to BH-3 mimetics was found in the terminal S3 stage. BM-derived LSK cells did not show increased sensitivity to BH3-mimetics when cultured in the presence of cytokines (Fig. 6D). Under cytokine deprivation, they became slightly sensitive to BCL-X_L_ and MCL-1 inhibition (Fig. 6E). Based on the promising in vitro effects, we used the BCL-X_L_ inhibitor A1155463 in vivo (Fig. 7A). Treatment for 28 days resulted in significant reduction of the spleen size in S2 mice (Fig. 7B). Analysis of all hematopoietic cells revealed significant decrease in monocytes and neutrophils but also HSPCs and erythroid progenitors in the spleen of treated mice (Fig. 7C–G), along with reduced infiltration in the blood and lungs (Fig. 7E). Interestingly, no effects were observed in BM or liver (Fig. 7D, E).

## Discussion

Our study aimed to characterize and target BCL-2-regulated apoptosis signaling, with the ultimate goal of identifying novel therapeutic approaches for high-risk JMML. Constitutively active RAS signaling, downstream of SHP2, was shown to exert anti-apoptotic effects [34, 35]. In hematopoietic cells, SHP2^D61Y^ or SHP2^E76K^ mutants were shown to have increased viability by upregulation of BCL-2 and BCL-X_L_ and downregulation of BIM. Similarly, *Ptpn11*^*E76K/+*^ transformed erythroleukemic TF-1 cells showed BCL-X_L_ upregulation [45]. Here, we compare different primary cell types at the same time, using a mouse model reminiscent of JMML and different culture conditions. We found that oncogenic SHP2^D61Y^ conferred survival advantages specifically to myeloid cells but not to HSPCs, despite high *Ptpn11* mRNA levels in LSK cells. Survival advantages were accompanied by BCL-X_L_ and/or MCL-1 upregulation in *Ptpn11* mutant myeloid cells and reversed by inhibition of the SHP2 downstream signaling, establishing a direct link between the oncogene and these anti-apoptotic proteins. This suggests that apoptosis resistance, alongside increased proliferation and differentiation, drives SHP2 myeloproliferation [13, 15]. The increased activation of RAS signaling in mature but not immature cells may explain why *PTPN11* mutations, when acquired as a first hit, lead to differentiating myelomonocytic leukemia rather than acute myeloid leukemia. It raises, however, the question, of why PTPN11 and/or KRAS mutations in HSCs allow relapse after transplantation. Most likely, PTPN11 and/or KRAS mutated HSCs acquire features providing selective fitness not linked to cell death signaling (i.e. immune escape mechanisms, metabolic fitness or others). Understanding the specific reasons for higher KRAS signaling in mature cells and the selective fitness of HSCs requires further detailed studies of the differentiation process, the regulatory networks involved, and the functional demands placed on both immature and mature cells.

In monocytes, both BCL-X_L_ and MCL-1 expression were directly linked to SHP2^D61Y^ but regulated differently: BCL-X_L_ expression was regulated by both MEK and RUNX1, whereas MCL-1 was regulated by MEK alone. In neutrophils, no clear link between SHP2^D61Y^ and the anti-apoptotic proteins was established. In addition, cell death decisions were affected by extracellular signals, which increased survival even in WT bystander cells. It was shown earlier that an abnormal microenvironment contributes to myeloproliferation in JMML mouse models, with CCL3/MIP-1 α playing an important role [46–48]. Here, we identified BCL-X_L_ as a novel player in this setting.

Our model revealed a cell type-specific “BCL-2 protein addiction”, where BCL-X_L_ and BCL-2 expression were essential for monocyte survival, whereas MCL-1 was crucial for neutrophils. Further analysis is needed to elucidate the regulation and engagement of these proteins. Differences in SHP2 signaling itself (i.e. activation levels and abundance of downstream signaling components), and other cell type-specific pathways, such as lineage-specific transcription factors, may influence the expression of BCL-2 family members. Potential candidates include NFkB and ETS, which were shown to influence BCL-2 protein expression, or the master transcription factors for myelomonocytic differentiation GATA2, PU.1 and GFI1 [49–51]. Similarly, the abundance of pro-apoptotic BH3-only proteins activated by developmental cues may differ between monocytes and granulocytes. Transcriptomic analyses and co-immunoprecipitation assays using highly purified cell populations will be necessary to fully delineate the apoptosis signaling pathways for each cell type.

Interestingly, apoptosis resistance increased during disease progression, with the most resistant granulocytes and monocytes at the lethal leukemia stage. Since there was no evidence of clonal evolution, we hypothesize that this is due to a gradual expansion of *Ptpn11*-mutant cells following polyI:C induction, combined with their selective advantages. However, we observed a strong downregulation of pro-apoptotic BH3-only and effector BCL-2 proteins in the late disease stage. At the same time, the response to BH3-mimetics changed, with a newly acquired BCL-2 dependency at the late disease stage. Most likely, niche-derived signals influence not only the abundance of BCL-X_L_ but also of pro-apoptotic proteins. Along this line, apoptosis resistance could be transferred to bystander WT cells in vivo but was not maintained in culture, suggesting a complex interplay of cell types and soluble factors contributing to apoptosis resistance. Indeed, several proteins with known anti-apoptotic function in granulocytes (i.e., CD74, TNFα, and ITGAM/CD11b) [52–55] were enriched in the microenvironment of leukemic mice. While these signals may cooperate to increase the viability of bystander cells in vivo, oncogene-driven cell-intrinsic signals are sufficient to ensure the survival of leukemic SHP2^D61Y^ cells.

Finally, we asked whether JMML could be cured by the BCL-X_L_ inhibitor, based on our in vitro data. Treatment significantly reduced leukemic burden and interestingly affected cells not sensitive in vitro (i.e HSPcs and erythroid progenitors). Again, this supports the hypothesis that niche-derived signals change apoptosis signaling in vivo [46–48] and highlights the need for disease-relevant in vivo models. Not unexpectedly, BCL-X_L_ inhibition used as monotherapy did not cure the mice. In patient-derived JMML cells, we recently identified synergistic effects of BCL-X_L_ and MCL-1 inhibitors. Along this line, azacitidine reduced MCL-1 levels, explaining its synergy with BCL-X_L_ inhibition in PDX mice [56]. Alternatively, therapeutic modulation of the microenvironment might increase the susceptibility of JMML cells towards BCL-x_L_ inhibition. Future strategies could focus on disrupting LSK-niche interactions using small molecule inhibitors, integrin blockers to modulate ECM dynamics, or interventions targeting niche-derived survival cytokines. Anti-CXCR4 therapies, such as plerixafor, used in AML [57] to disrupt interactions with the bone marrow microenvironment, could be explored as a potential treatment in JMML.

The JMML PDX and genetically modified mouse models provide complementary insights into RAS dysregulation on apoptotic signaling. While the PDX model excels in translational relevance by mimicking key JMML features in patients (including methylation signatures), its reliance on immunodeficient mice limits the study of RAS effects on the complete hematopoietic and immune system. The genetically modified mouse model used in this study, in contrast, preserves immune interactions and enables detailed investigation of leukemogenesis in an immunocompetent setting, offering a more comprehensive view of disease progression on the full hematopoietic compartment. A more comprehensive understanding of apoptosis and other cell death forms, such as necroptosis and pyroptosis [58] in both models are crucial for harnessing cell death in therapeutic applications and to develop effective combination therapies.

## Supplementary information

Supplementary material

WB_original pictures.

## Data Availability

The mass spectrometry proteomics data have been deposited to the ProteomeXchange Consortium via the PRIDE (PMID 34723319) partner repository with the dataset identifier PXD055845. The next-generation sequencing data is deposited at NCBI GEO (GSE277667). All other data generated and analyzed during this study are included in this published article and supplementary information.
