# Supplementary material for: Oncogenic and microenvironmental signals drive cell type specific apoptosis resistance in juvenile myelomonocytic leukemia
Source: Cell Death Dis. 2025 Mar 8;16(1):165. doi: 10.1038/s41419-025-07479-2 (PMC11890777; doi:10.1038/s41419-025-07479-2)
Supplement: Supplementary file 1 — Supplementary material [file 41419_2025_7479_MOESM1_ESM.pptx]

## Slide 1
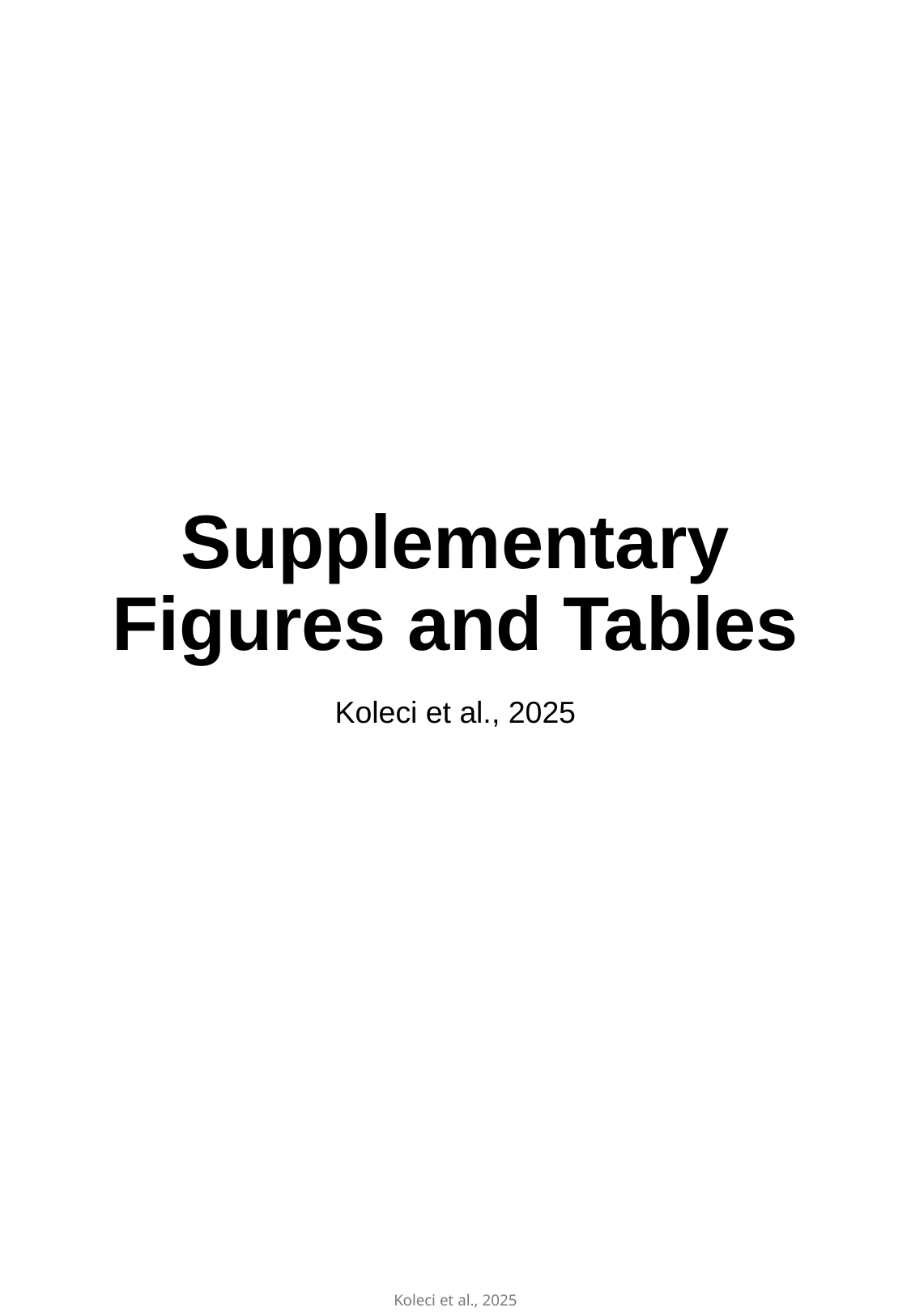

# Supplementary Figures and Tables
Koleci et al., 2025
Koleci et al., 2025

## Slide 2
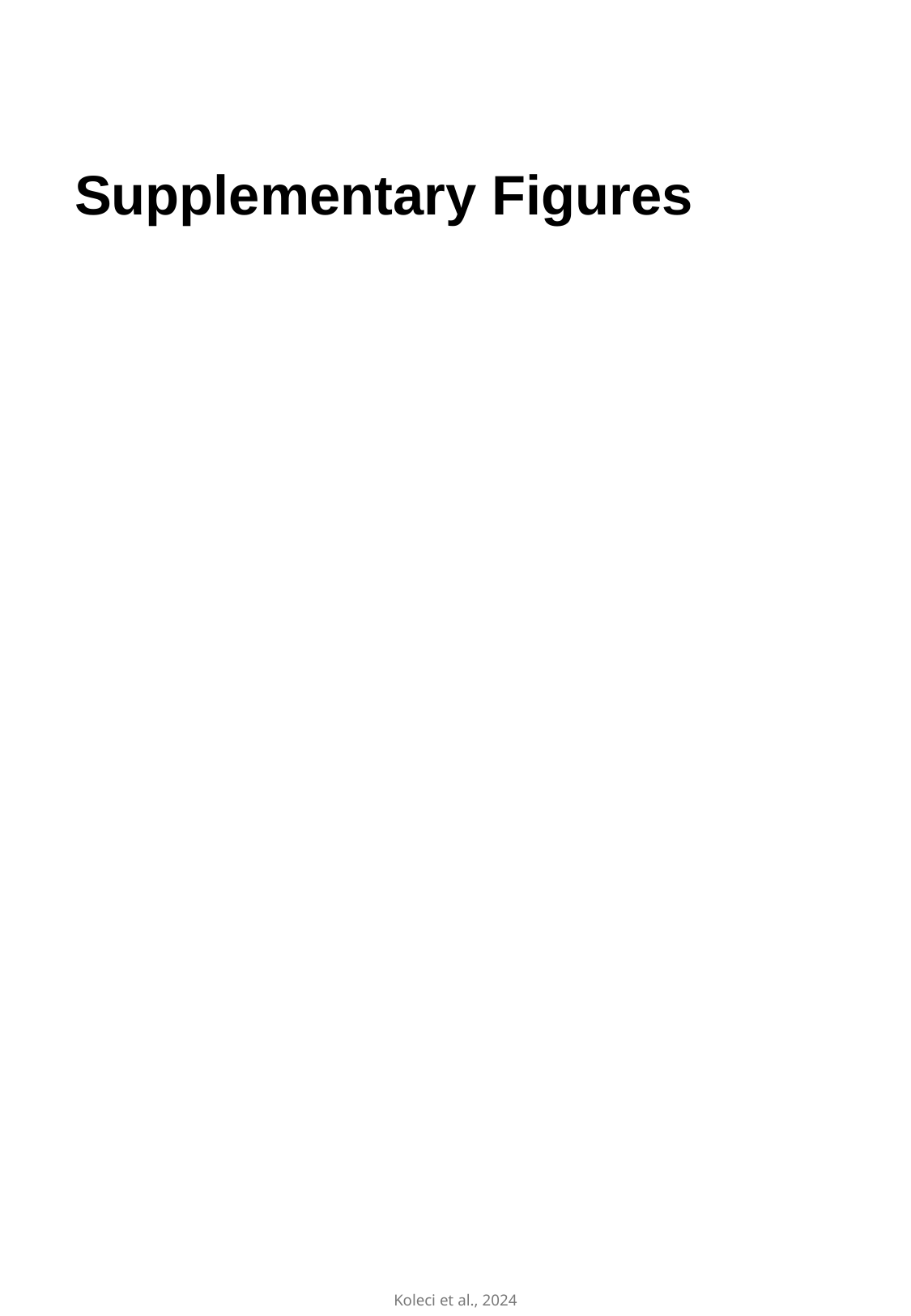

# Supplementary Figures
Koleci et al., 2024

## Slide 3
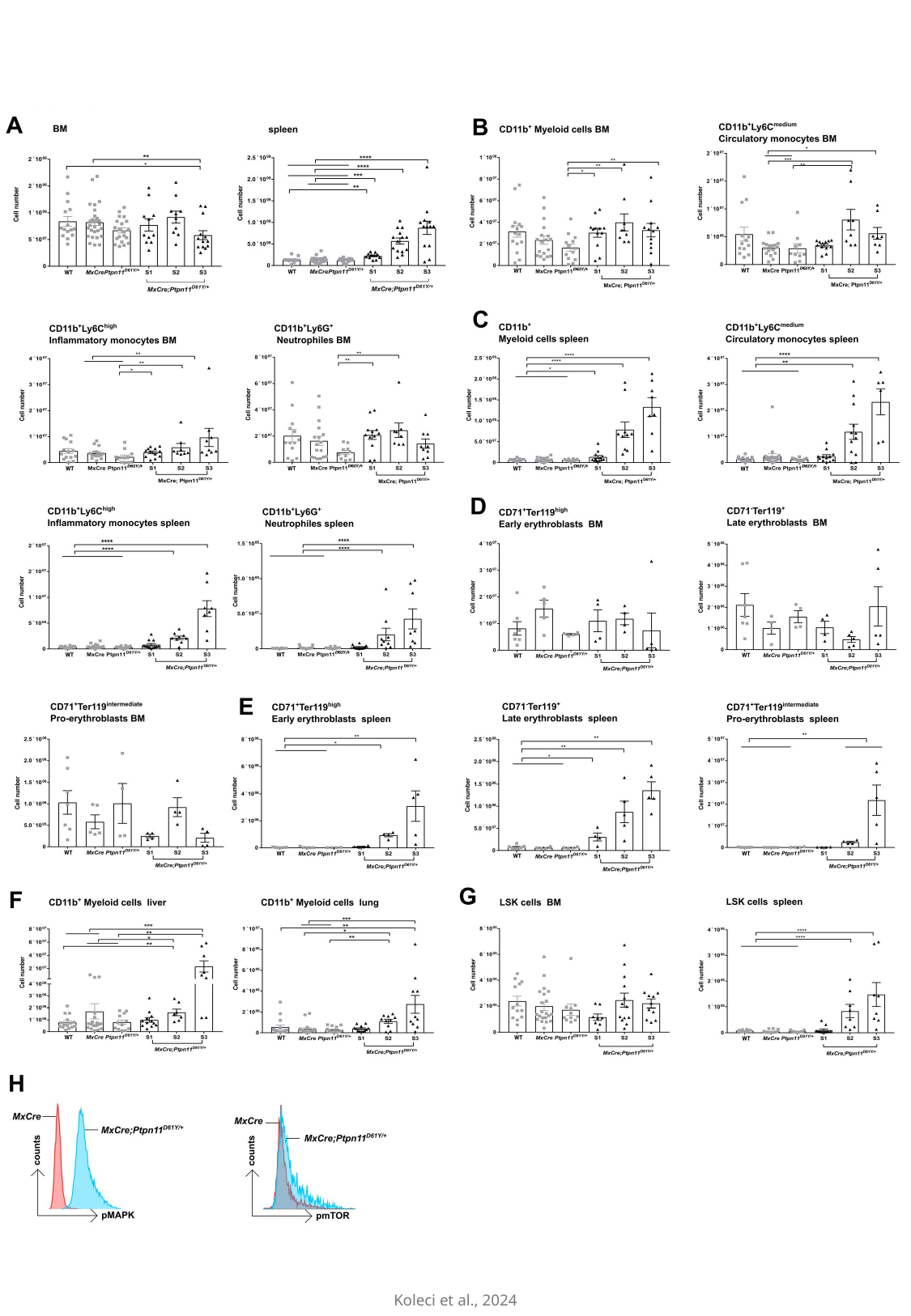

Koleci et al., 2024

## Slide 4
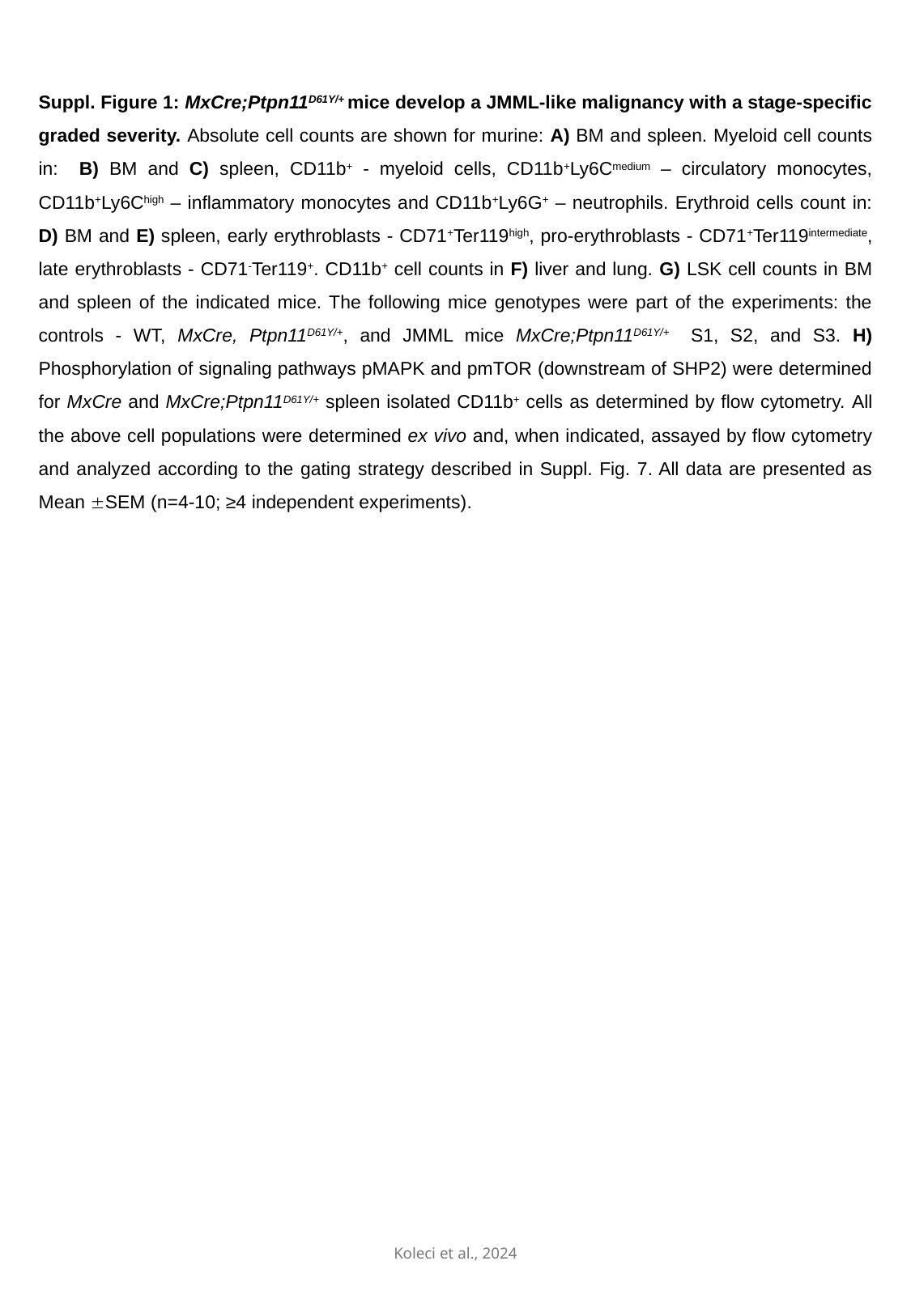

Suppl. Figure 1: MxCre;Ptpn11D61Y/+ mice develop a JMML-like malignancy with a stage-specific graded severity. Absolute cell counts are shown for murine: A) BM and spleen. Myeloid cell counts in: B) BM and C) spleen, CD11b+ - myeloid cells, CD11b+Ly6Cmedium – circulatory monocytes, CD11b+Ly6Chigh – inflammatory monocytes and CD11b+Ly6G+ – neutrophils. Erythroid cells count in: D) BM and E) spleen, early erythroblasts - CD71+Ter119high, pro-erythroblasts - CD71+Ter119intermediate, late erythroblasts - CD71-Ter119+. CD11b+ cell counts in F) liver and lung. G) LSK cell counts in BM and spleen of the indicated mice. The following mice genotypes were part of the experiments: the controls - WT, MxCre, Ptpn11D61Y/+, and JMML mice MxCre;Ptpn11D61Y/+ S1, S2, and S3. H) Phosphorylation of signaling pathways pMAPK and pmTOR (downstream of SHP2) were determined for MxCre and MxCre;Ptpn11D61Y/+ spleen isolated CD11b+ cells as determined by flow cytometry. All the above cell populations were determined ex vivo and, when indicated, assayed by flow cytometry and analyzed according to the gating strategy described in Suppl. Fig. 7. All data are presented as Mean ±SEM (n=4-10; ≥4 independent experiments).
Koleci et al., 2024

## Slide 5
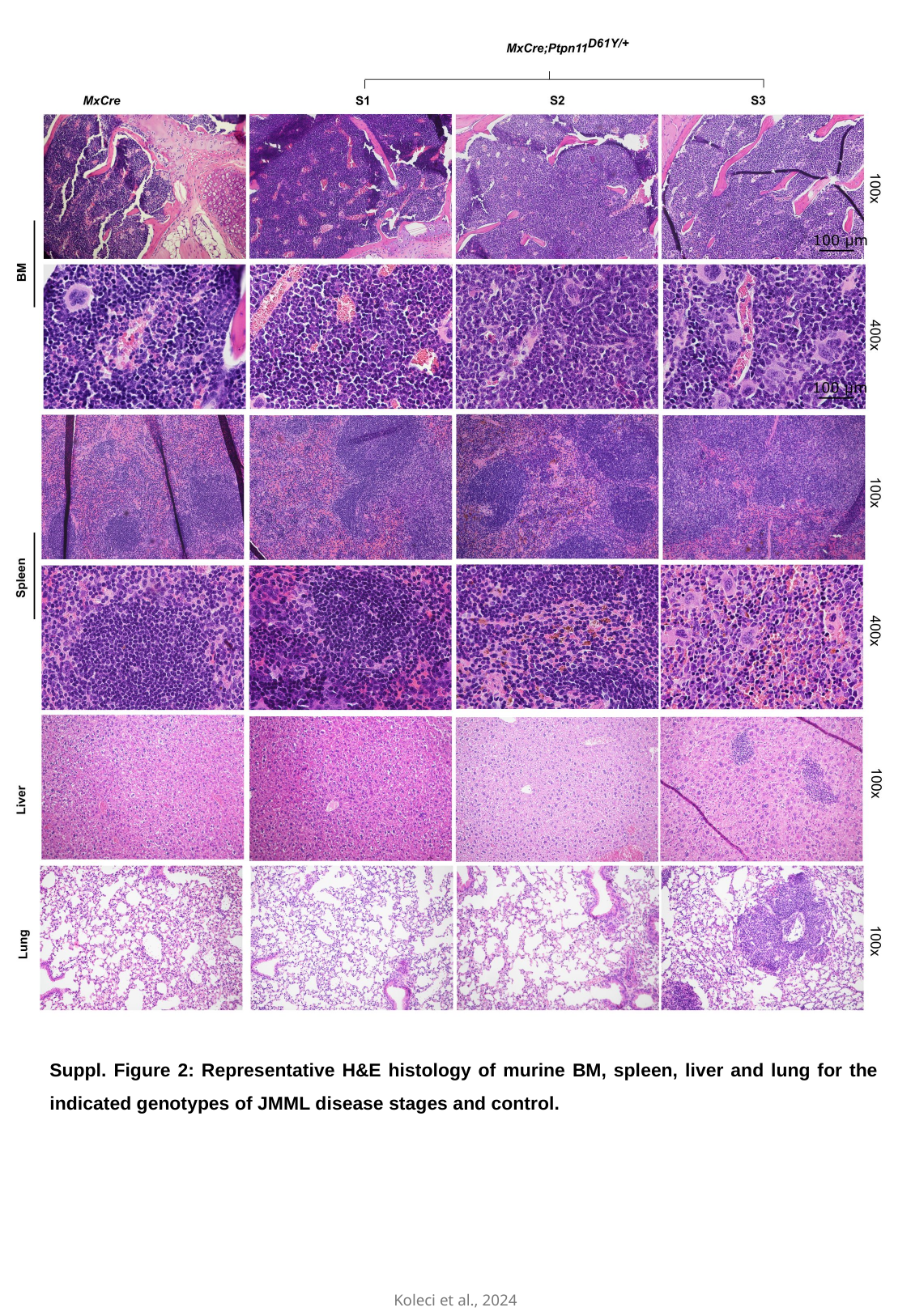

Suppl. Figure 2: Representative H&E histology of murine BM, spleen, liver and lung for the indicated genotypes of JMML disease stages and control.
Koleci et al., 2024

## Slide 6
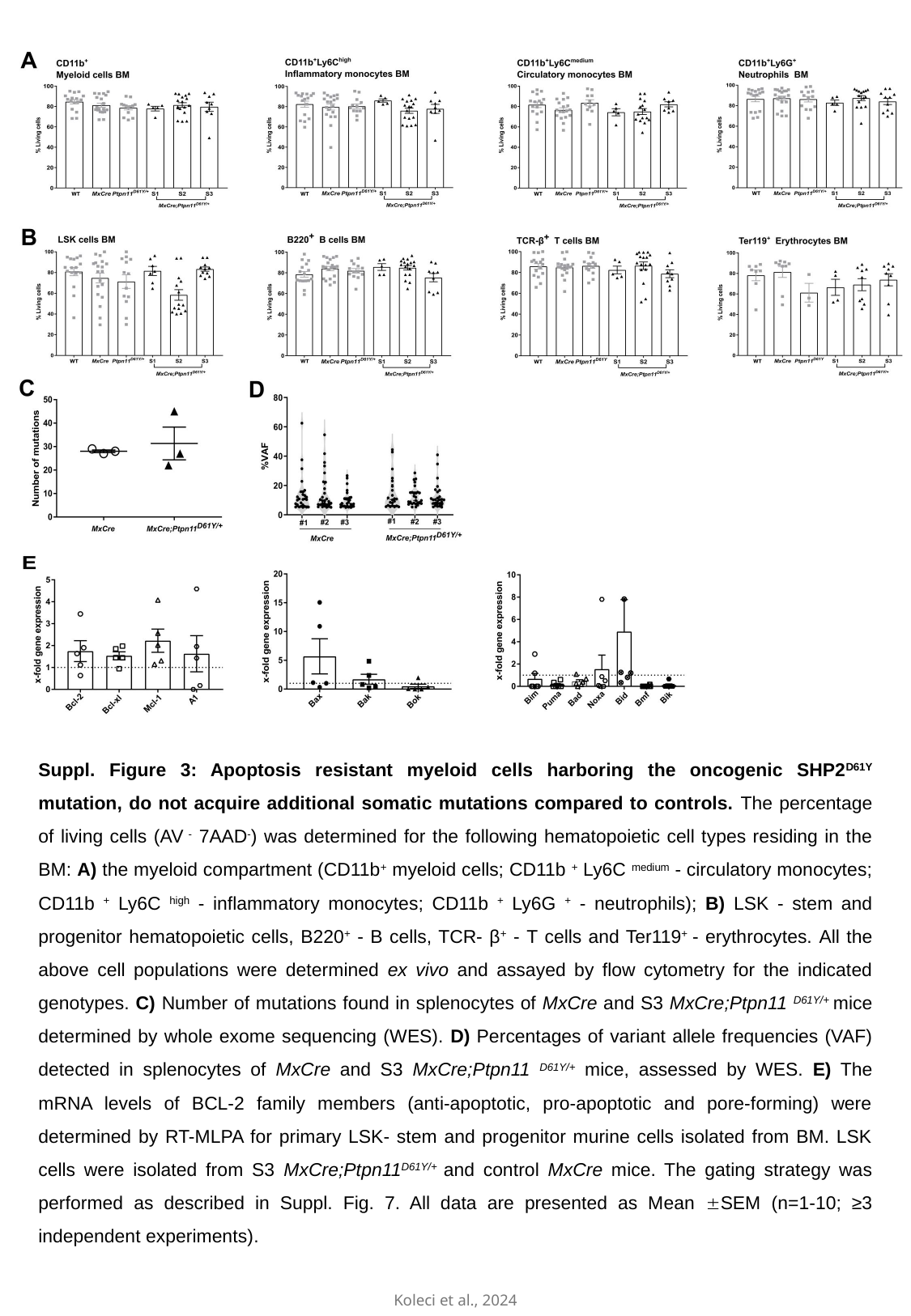

Suppl. Figure 3: Apoptosis resistant myeloid cells harboring the oncogenic SHP2D61Y mutation, do not acquire additional somatic mutations compared to controls. The percentage of living cells (AV - 7AAD-) was determined for the following hematopoietic cell types residing in the BM: A) the myeloid compartment (CD11b+ myeloid cells; CD11b + Ly6C medium - circulatory monocytes; CD11b + Ly6C high - inflammatory monocytes; CD11b + Ly6G + - neutrophils); B) LSK - stem and progenitor hematopoietic cells, B220+ - B cells, TCR- β+ - T cells and Ter119+ - erythrocytes. All the above cell populations were determined ex vivo and assayed by flow cytometry for the indicated genotypes. C) Number of mutations found in splenocytes of MxCre and S3 MxCre;Ptpn11 D61Y/+ mice determined by whole exome sequencing (WES). D) Percentages of variant allele frequencies (VAF) detected in splenocytes of MxCre and S3 MxCre;Ptpn11 D61Y/+ mice, assessed by WES. E) The mRNA levels of BCL-2 family members (anti-apoptotic, pro-apoptotic and pore-forming) were determined by RT-MLPA for primary LSK- stem and progenitor murine cells isolated from BM. LSK cells were isolated from S3 MxCre;Ptpn11D61Y/+ and control MxCre mice. The gating strategy was performed as described in Suppl. Fig. 7. All data are presented as Mean ±SEM (n=1-10; ≥3 independent experiments).
Koleci et al., 2024

## Slide 7
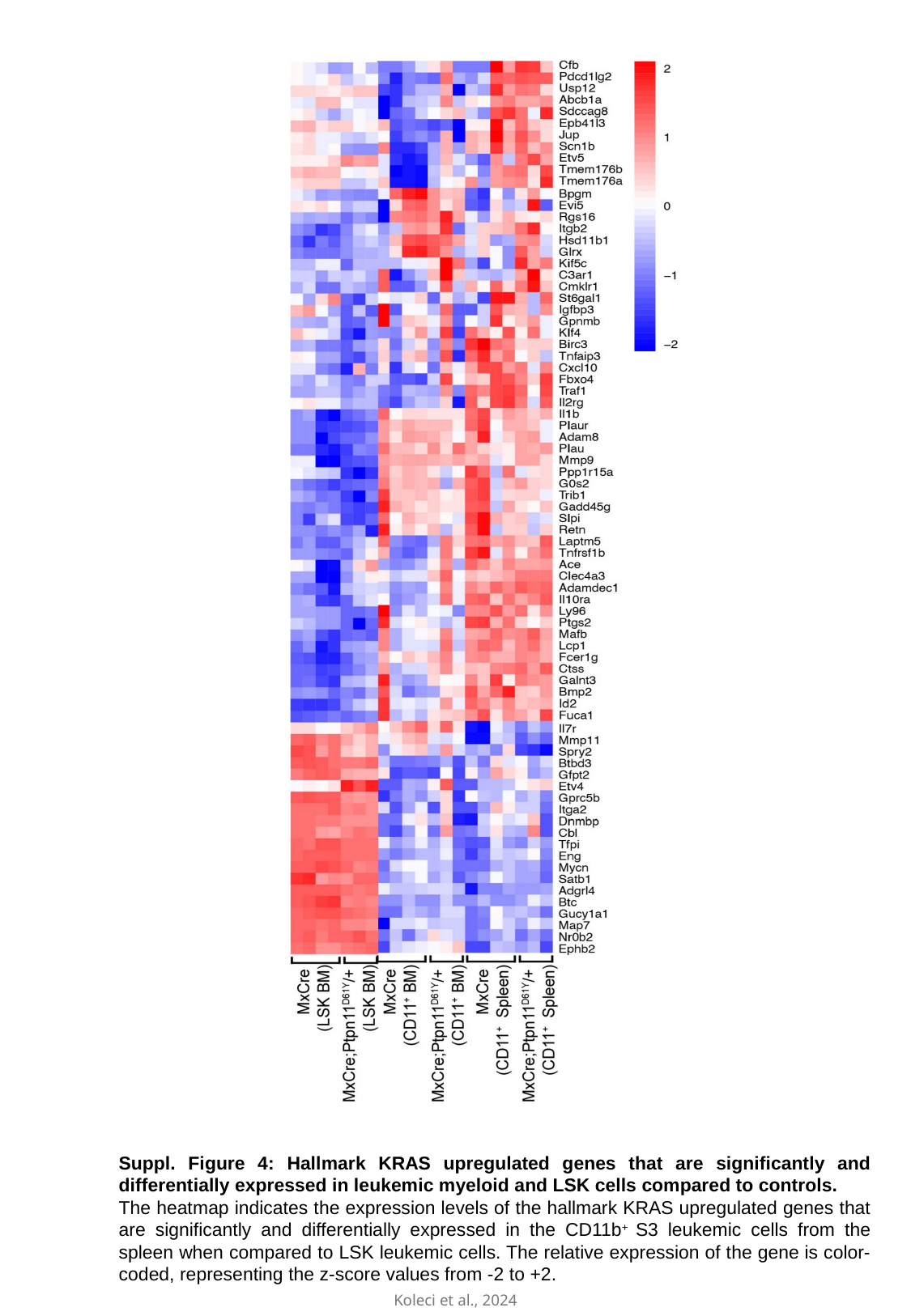

Suppl. Figure 4: Hallmark KRAS upregulated genes that are significantly and differentially expressed in leukemic myeloid and LSK cells compared to controls.
The heatmap indicates the expression levels of the hallmark KRAS upregulated genes that are significantly and differentially expressed in the CD11b+ S3 leukemic cells from the spleen when compared to LSK leukemic cells. The relative expression of the gene is color-coded, representing the z-score values from -2 to +2.
Koleci et al., 2024

## Slide 8
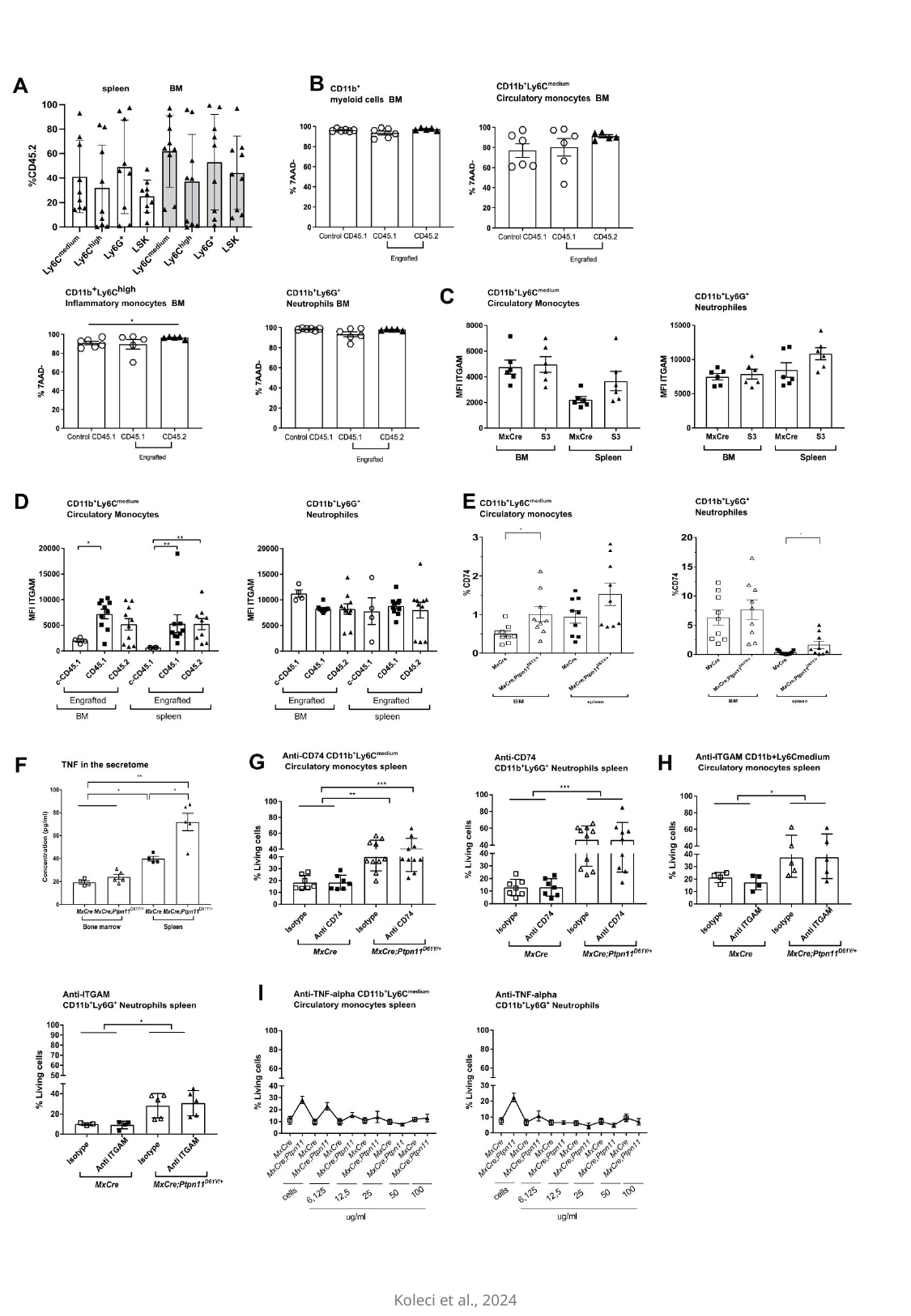

Koleci et al., 2024

## Slide 9
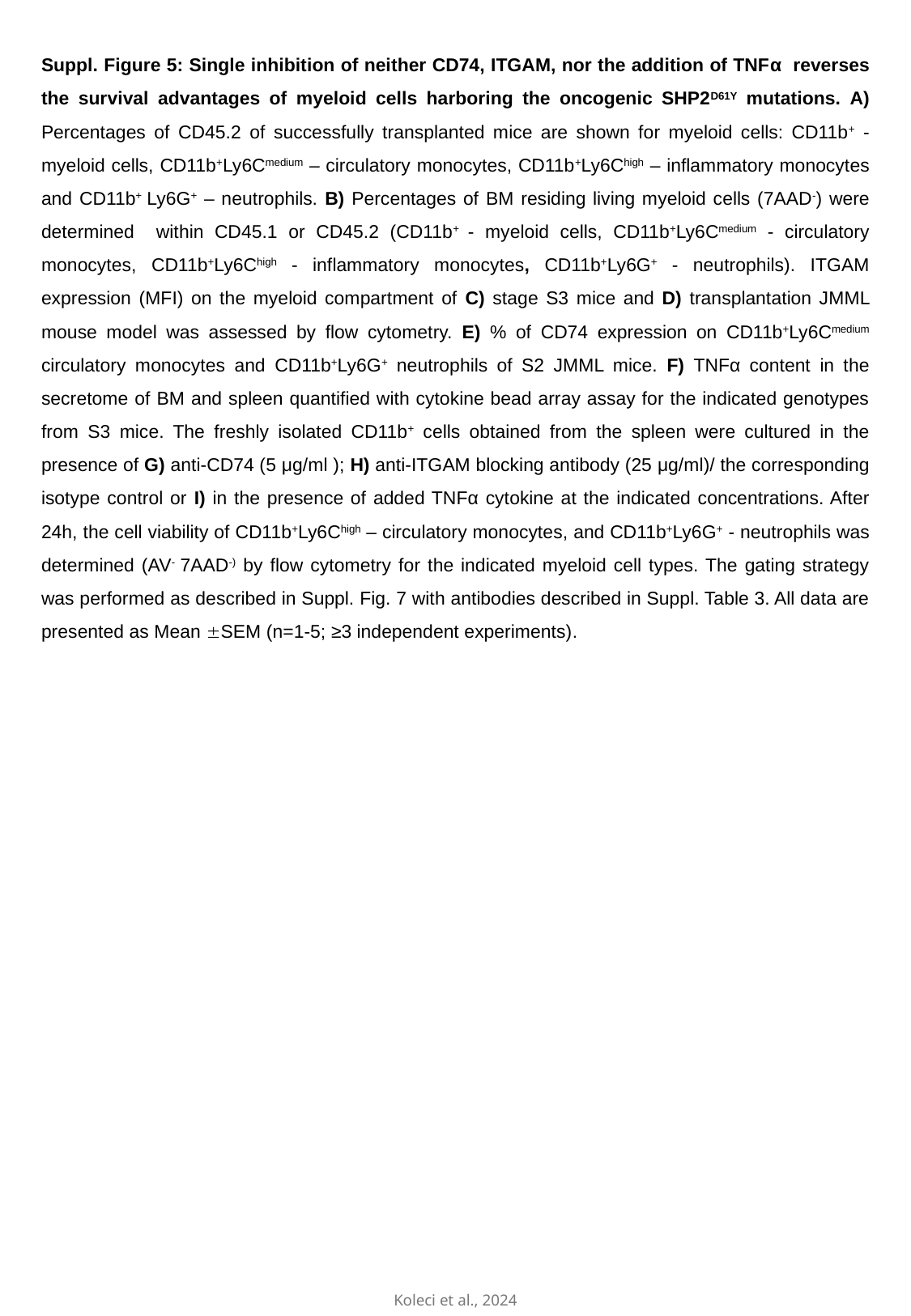

Suppl. Figure 5: Single inhibition of neither CD74, ITGAM, nor the addition of TNFα reverses the survival advantages of myeloid cells harboring the oncogenic SHP2D61Y mutations. A) Percentages of CD45.2 of successfully transplanted mice are shown for myeloid cells: CD11b+ - myeloid cells, CD11b+Ly6Cmedium – circulatory monocytes, CD11b+Ly6Chigh – inflammatory monocytes and CD11b+ Ly6G+ – neutrophils. B) Percentages of BM residing living myeloid cells (7AAD-) were determined within CD45.1 or CD45.2 (CD11b+ - myeloid cells, CD11b+Ly6Cmedium - circulatory monocytes, CD11b+Ly6Chigh - inflammatory monocytes, CD11b+Ly6G+ - neutrophils). ITGAM expression (MFI) on the myeloid compartment of C) stage S3 mice and D) transplantation JMML mouse model was assessed by flow cytometry. E) % of CD74 expression on CD11b+Ly6Cmedium circulatory monocytes and CD11b+Ly6G+ neutrophils of S2 JMML mice. F) TNFα content in the secretome of BM and spleen quantified with cytokine bead array assay for the indicated genotypes from S3 mice. The freshly isolated CD11b+ cells obtained from the spleen were cultured in the presence of G) anti-CD74 (5 μg/ml ); H) anti-ITGAM blocking antibody (25 μg/ml)/ the corresponding isotype control or I) in the presence of added TNFα cytokine at the indicated concentrations. After 24h, the cell viability of CD11b+Ly6Chigh – circulatory monocytes, and CD11b+Ly6G+ - neutrophils was determined (AV- 7AAD-) by flow cytometry for the indicated myeloid cell types. The gating strategy was performed as described in Suppl. Fig. 7 with antibodies described in Suppl. Table 3. All data are presented as Mean ±SEM (n=1-5; ≥3 independent experiments).
Koleci et al., 2024

## Slide 10
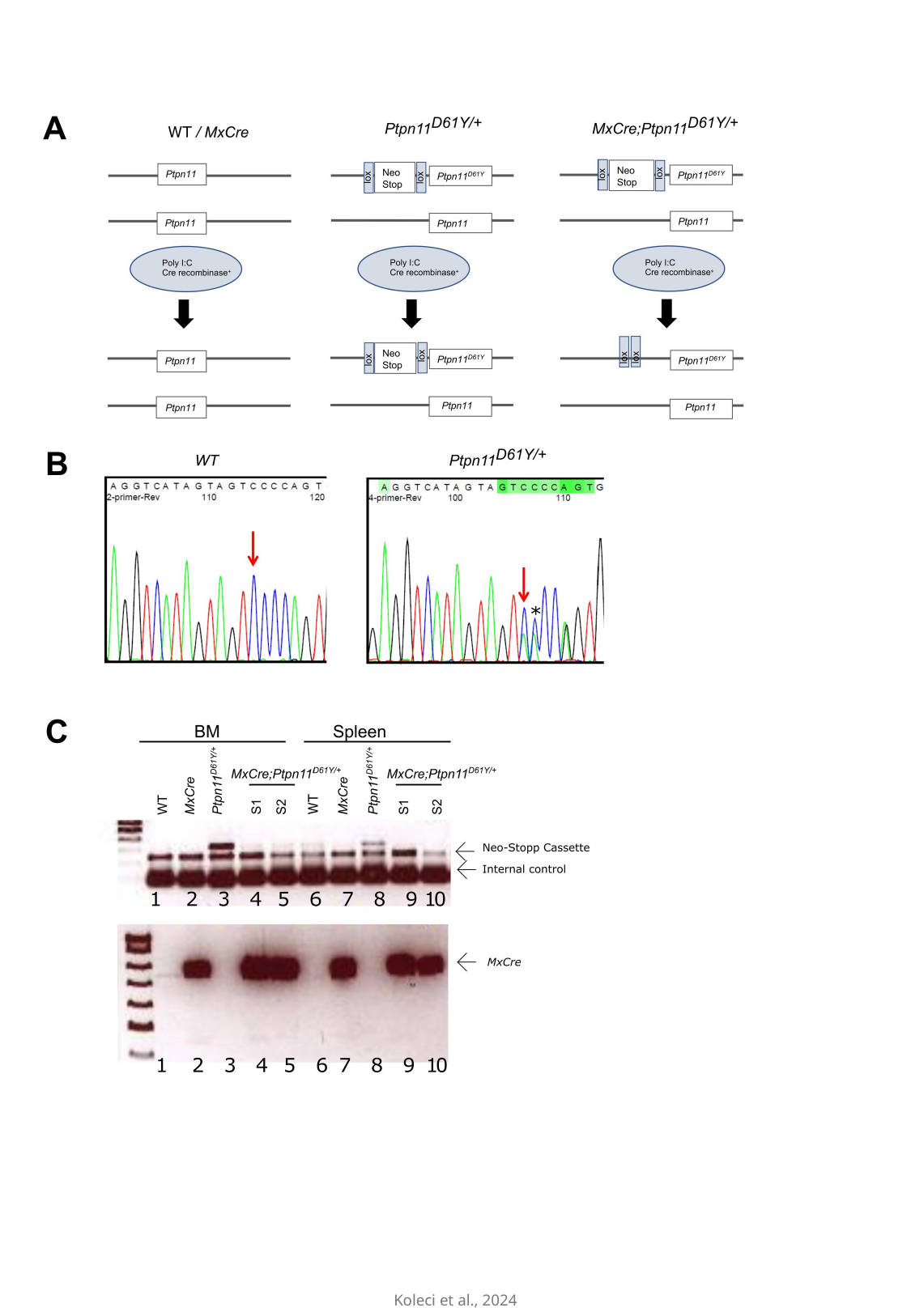

Koleci et al., 2024

## Slide 11
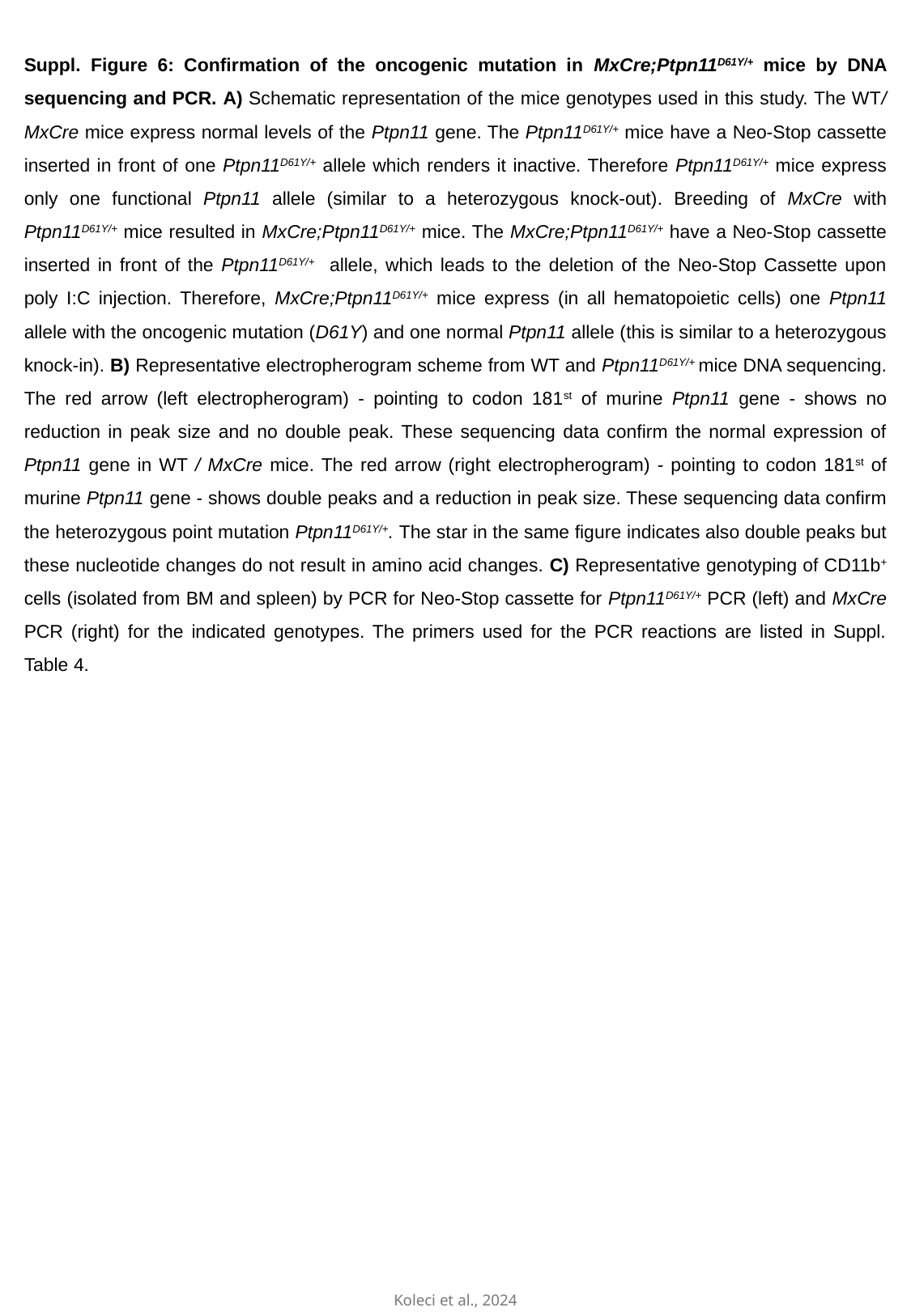

Suppl. Figure 6: Confirmation of the oncogenic mutation in MxCre;Ptpn11D61Y/+ mice by DNA sequencing and PCR. A) Schematic representation of the mice genotypes used in this study. The WT/ MxCre mice express normal levels of the Ptpn11 gene. The Ptpn11D61Y/+ mice have a Neo-Stop cassette inserted in front of one Ptpn11D61Y/+ allele which renders it inactive. Therefore Ptpn11D61Y/+ mice express only one functional Ptpn11 allele (similar to a heterozygous knock-out). Breeding of MxCre with Ptpn11D61Y/+ mice resulted in MxCre;Ptpn11D61Y/+ mice. The MxCre;Ptpn11D61Y/+ have a Neo-Stop cassette inserted in front of the Ptpn11D61Y/+ allele, which leads to the deletion of the Neo-Stop Cassette upon poly I:C injection. Therefore, MxCre;Ptpn11D61Y/+ mice express (in all hematopoietic cells) one Ptpn11 allele with the oncogenic mutation (D61Y) and one normal Ptpn11 allele (this is similar to a heterozygous knock-in). B) Representative electropherogram scheme from WT and Ptpn11D61Y/+ mice DNA sequencing. The red arrow (left electropherogram) - pointing to codon 181st of murine Ptpn11 gene - shows no reduction in peak size and no double peak. These sequencing data confirm the normal expression of Ptpn11 gene in WT / MxCre mice. The red arrow (right electropherogram) - pointing to codon 181st of murine Ptpn11 gene - shows double peaks and a reduction in peak size. These sequencing data confirm the heterozygous point mutation Ptpn11D61Y/+. The star in the same figure indicates also double peaks but these nucleotide changes do not result in amino acid changes. C) Representative genotyping of CD11b+ cells (isolated from BM and spleen) by PCR for Neo-Stop cassette for Ptpn11D61Y/+ PCR (left) and MxCre PCR (right) for the indicated genotypes. The primers used for the PCR reactions are listed in Suppl. Table 4.
Koleci et al., 2024

## Slide 12
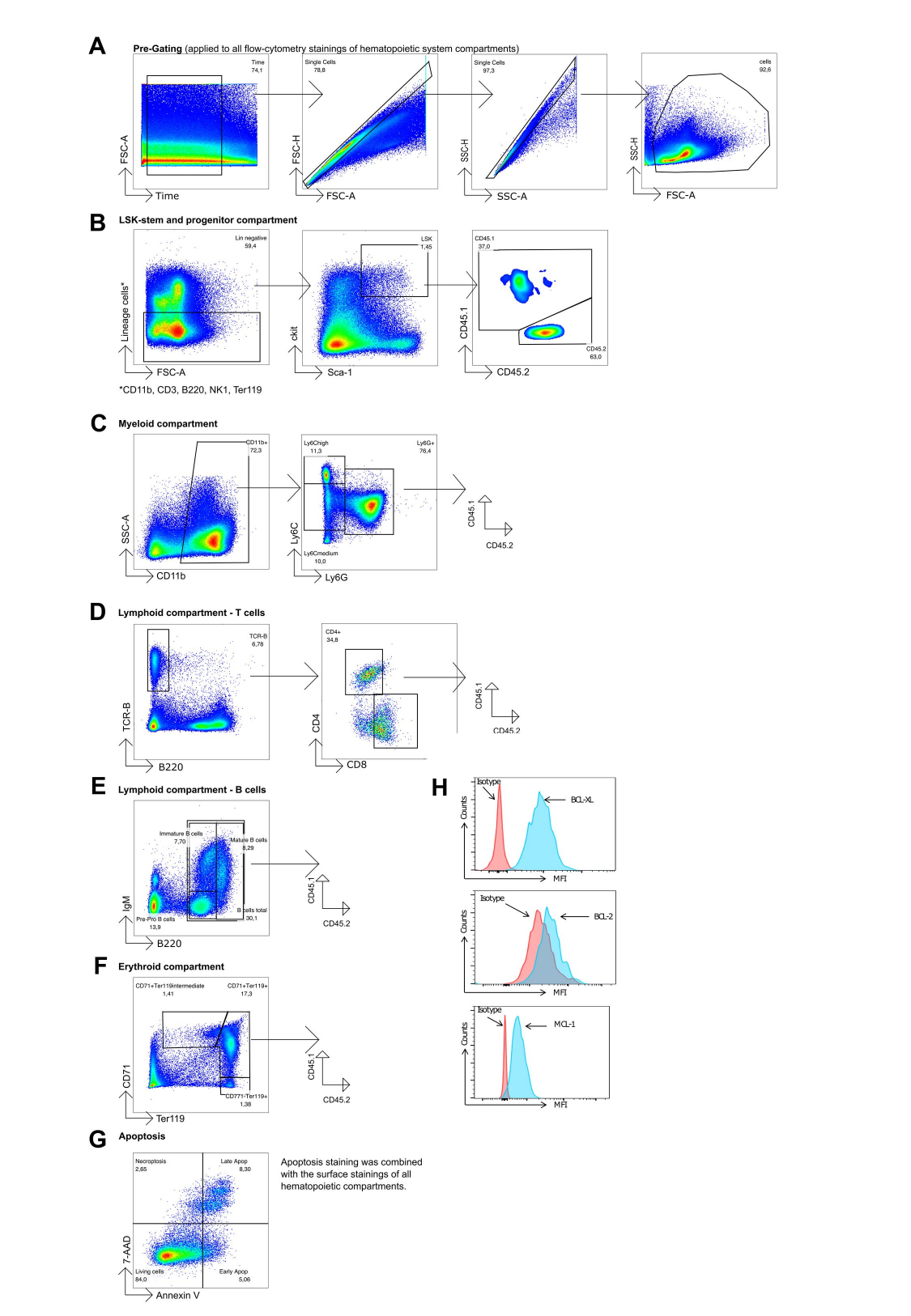

Koleci et al., 2024

## Slide 13
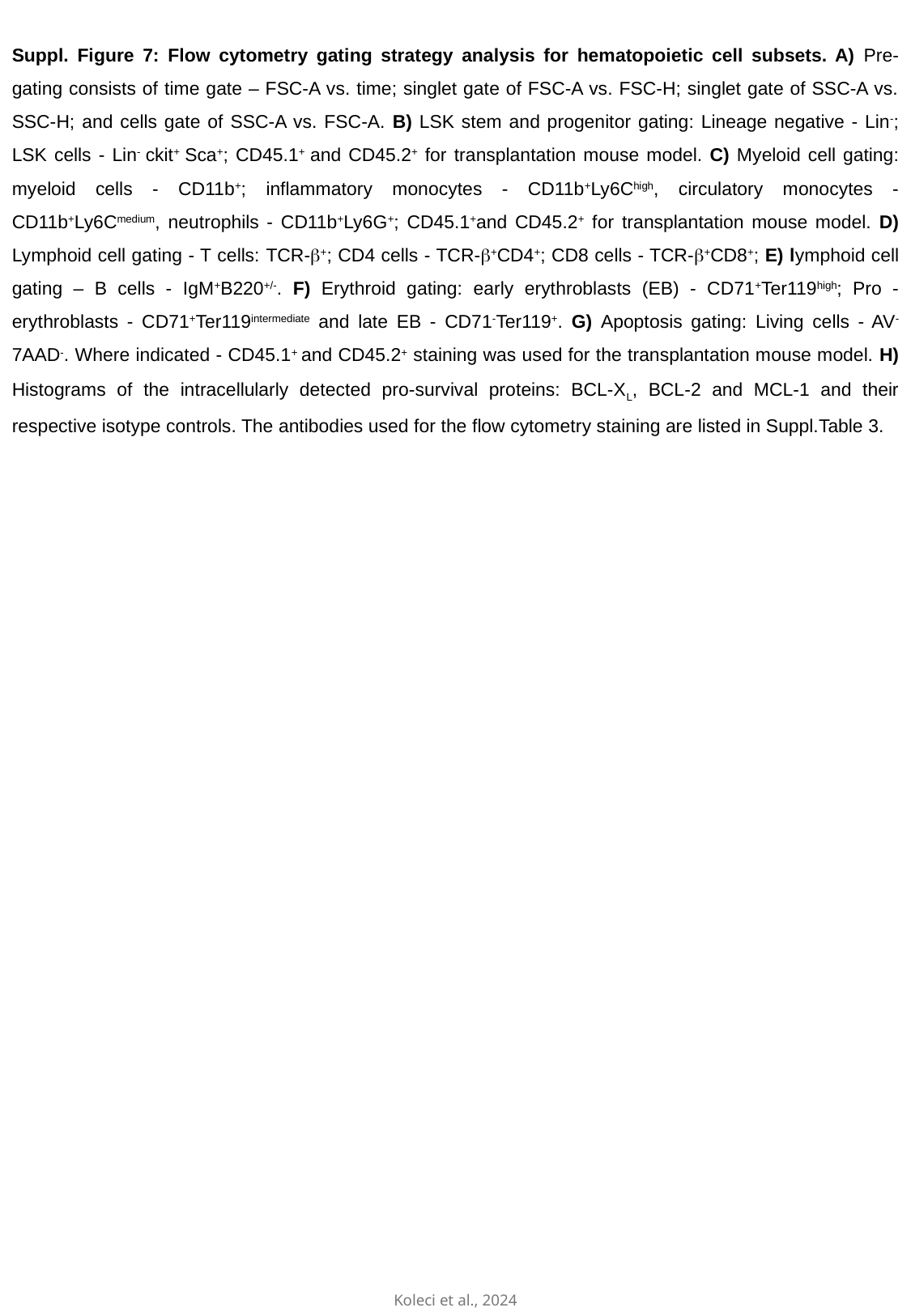

Suppl. Figure 7: Flow cytometry gating strategy analysis for hematopoietic cell subsets. A) Pre-gating consists of time gate – FSC-A vs. time; singlet gate of FSC-A vs. FSC-H; singlet gate of SSC-A vs. SSC-H; and cells gate of SSC-A vs. FSC-A. B) LSK stem and progenitor gating: Lineage negative - Lin-; LSK cells - Lin- ckit+ Sca+; CD45.1+ and CD45.2+ for transplantation mouse model. C) Myeloid cell gating: myeloid cells - CD11b+; inflammatory monocytes - CD11b+Ly6Chigh, circulatory monocytes - CD11b+Ly6Cmedium, neutrophils - CD11b+Ly6G+; CD45.1+and CD45.2+ for transplantation mouse model. D) Lymphoid cell gating - T cells: TCR-β+; CD4 cells - TCR-β+CD4+; CD8 cells - TCR-β+CD8+; E) lymphoid cell gating – B cells - IgM+B220+/-. F) Erythroid gating: early erythroblasts (EB) - CD71+Ter119high; Pro - erythroblasts - CD71+Ter119intermediate and late EB - CD71-Ter119+. G) Apoptosis gating: Living cells - AV-7AAD-. Where indicated - CD45.1+ and CD45.2+ staining was used for the transplantation mouse model. H) Histograms of the intracellularly detected pro-survival proteins: BCL-XL, BCL-2 and MCL-1 and their respective isotype controls. The antibodies used for the flow cytometry staining are listed in Suppl.Table 3.
Koleci et al., 2024

## Slide 14
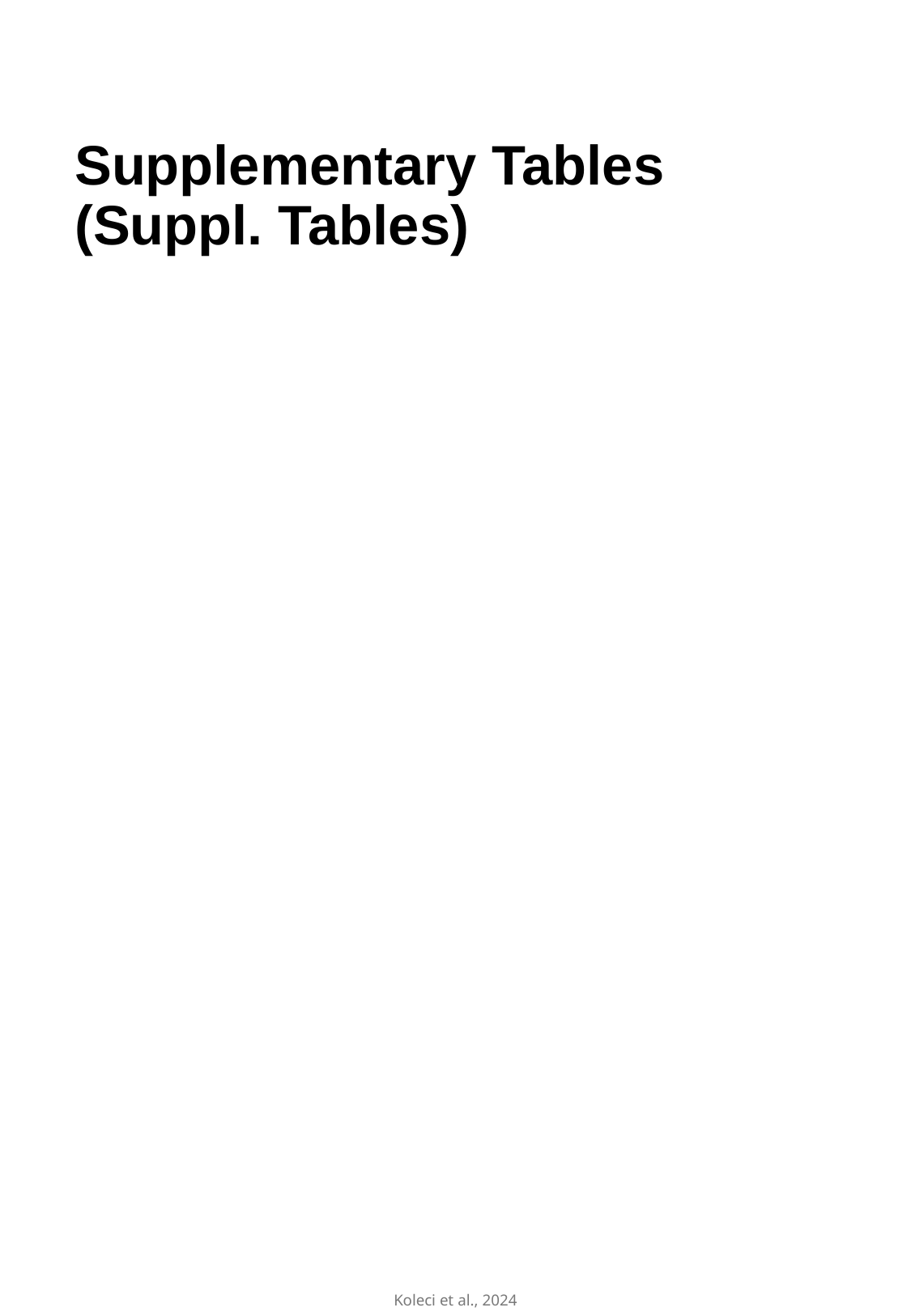

# Supplementary Tables (Suppl. Tables)
Koleci et al., 2024

## Slide 15
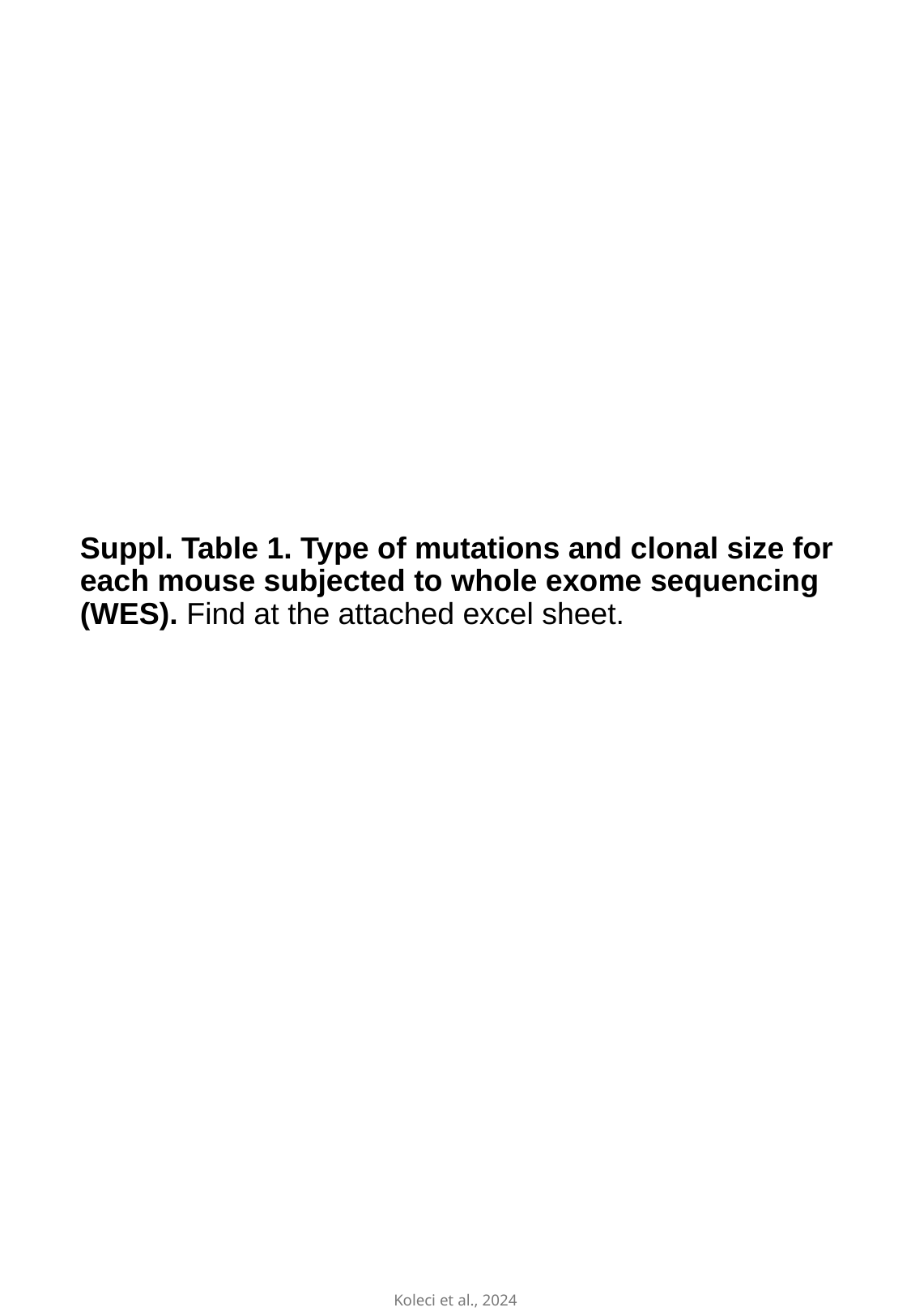

# Suppl. Table 1. Type of mutations and clonal size for each mouse subjected to whole exome sequencing (WES). Find at the attached excel sheet.
Koleci et al., 2024

## Slide 16
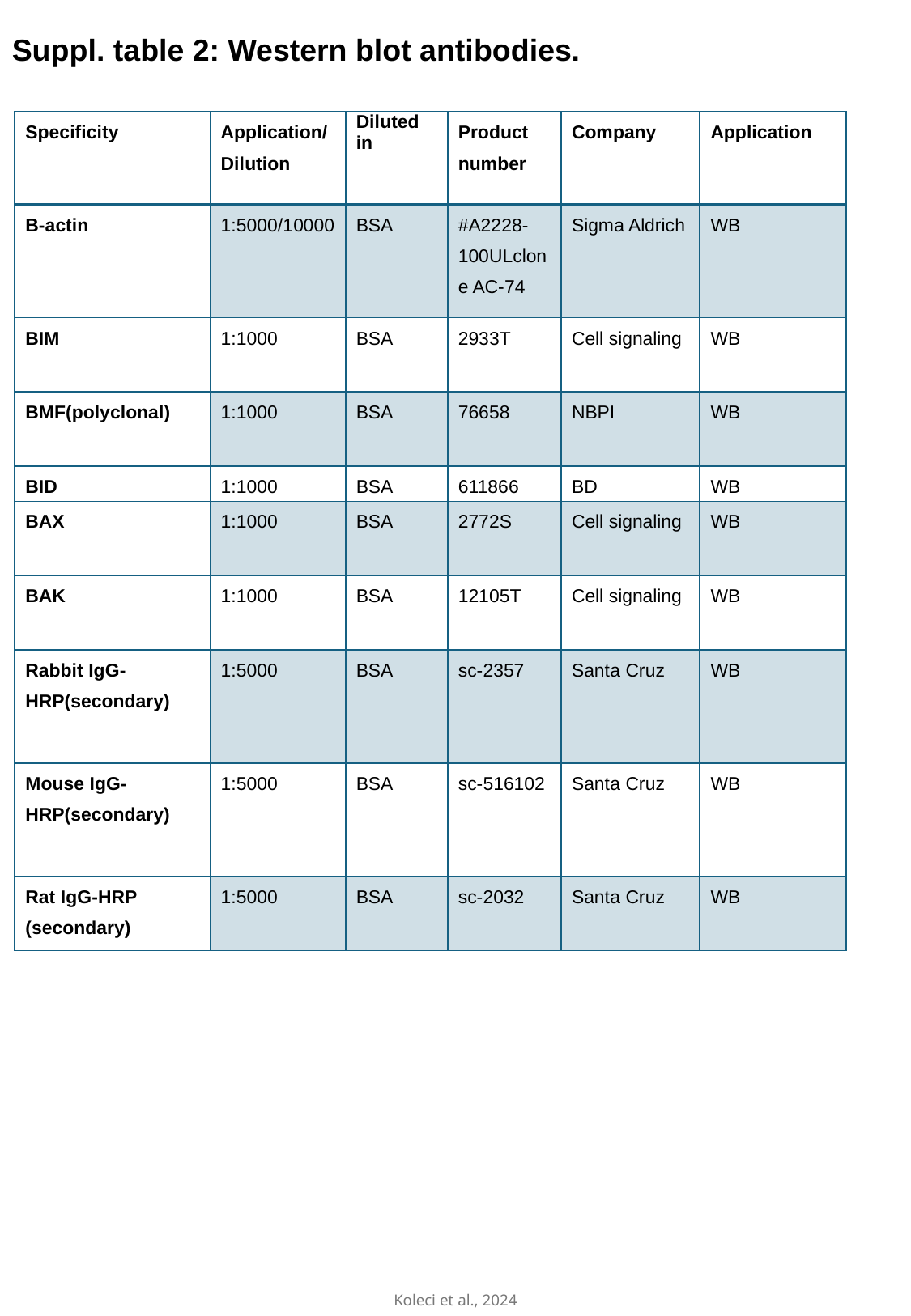

Suppl. table 2: Western blot antibodies.
| Specificity | Application/ Dilution | Diluted in | Product number | Company | Application |
| --- | --- | --- | --- | --- | --- |
| B-actin | 1:5000/10000 | BSA | #A2228-100ULclone AC-74 | Sigma Aldrich | WB |
| BIM | 1:1000 | BSA | 2933T | Cell signaling | WB |
| BMF(polyclonal) | 1:1000 | BSA | 76658 | NBPI | WB |
| BID | 1:1000 | BSA | 611866 | BD | WB |
| BAX | 1:1000 | BSA | 2772S | Cell signaling | WB |
| BAK | 1:1000 | BSA | 12105T | Cell signaling | WB |
| Rabbit IgG-HRP(secondary) | 1:5000 | BSA | sc-2357 | Santa Cruz | WB |
| Mouse IgG-HRP(secondary) | 1:5000 | BSA | sc-516102 | Santa Cruz | WB |
| Rat IgG-HRP (secondary) | 1:5000 | BSA | sc-2032 | Santa Cruz | WB |
Koleci et al., 2024

## Slide 17
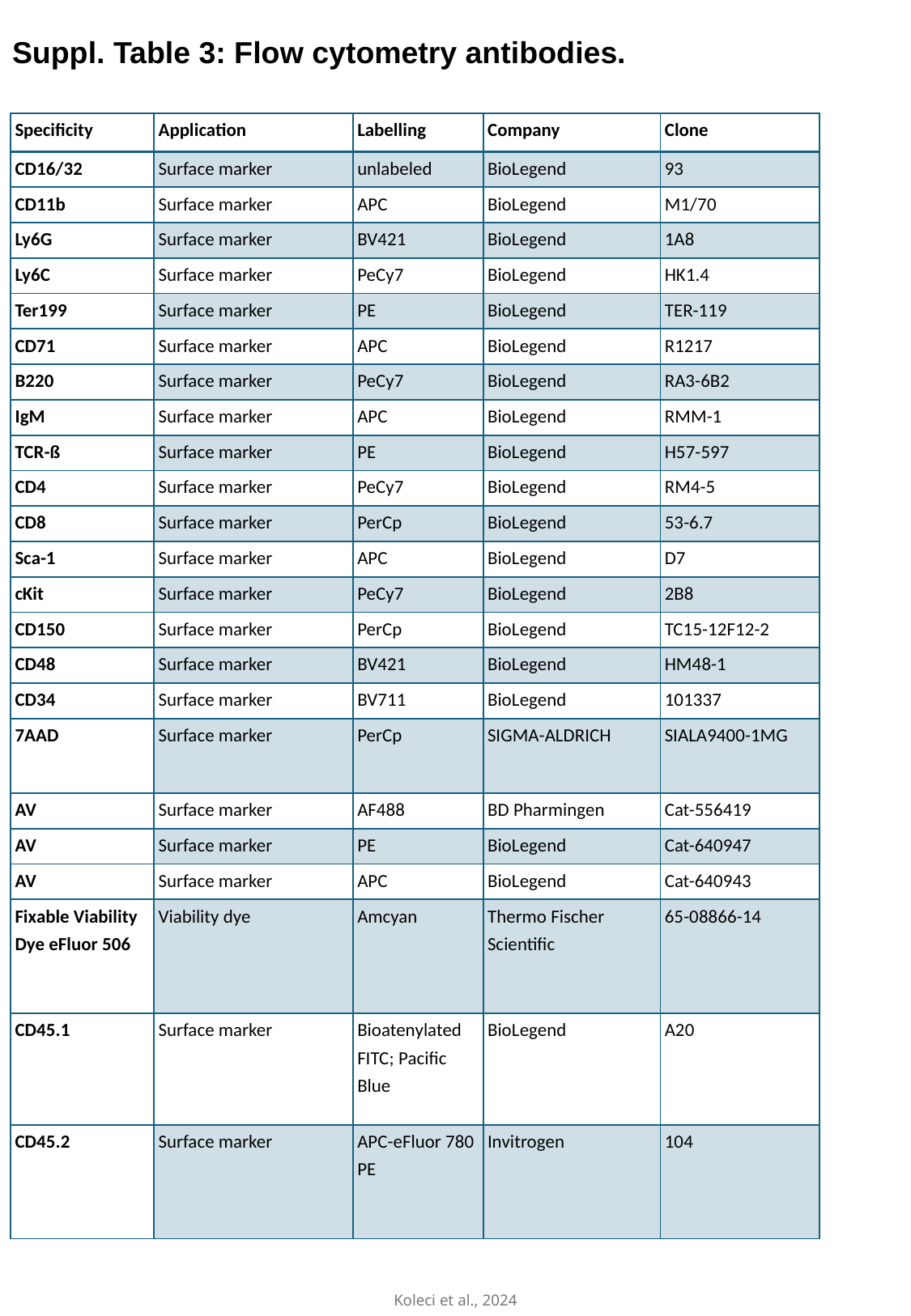

# Suppl. Table 3: Flow cytometry antibodies.
| Specificity | Application | Labelling | Company | Clone |
| --- | --- | --- | --- | --- |
| CD16/32 | Surface marker | unlabeled | BioLegend | 93 |
| CD11b | Surface marker | APC | BioLegend | M1/70 |
| Ly6G | Surface marker | BV421 | BioLegend | 1A8 |
| Ly6C | Surface marker | PeCy7 | BioLegend | HK1.4 |
| Ter199 | Surface marker | PE | BioLegend | TER-119 |
| CD71 | Surface marker | APC | BioLegend | R1217 |
| B220 | Surface marker | PeCy7 | BioLegend | RA3-6B2 |
| IgM | Surface marker | APC | BioLegend | RMM-1 |
| TCR-ß | Surface marker | PE | BioLegend | H57-597 |
| CD4 | Surface marker | PeCy7 | BioLegend | RM4-5 |
| CD8 | Surface marker | PerCp | BioLegend | 53-6.7 |
| Sca-1 | Surface marker | APC | BioLegend | D7 |
| cKit | Surface marker | PeCy7 | BioLegend | 2B8 |
| CD150 | Surface marker | PerCp | BioLegend | TC15-12F12-2 |
| CD48 | Surface marker | BV421 | BioLegend | HM48-1 |
| CD34 | Surface marker | BV711 | BioLegend | 101337 |
| 7AAD | Surface marker | PerCp | SIGMA-ALDRICH | SIALA9400-1MG |
| AV | Surface marker | AF488 | BD Pharmingen | Cat-556419 |
| AV | Surface marker | PE | BioLegend | Cat-640947 |
| AV | Surface marker | APC | BioLegend | Cat-640943 |
| Fixable Viability Dye eFluor 506 | Viability dye | Amcyan | Thermo Fischer Scientific | 65-08866-14 |
| CD45.1 | Surface marker | Bioatenylated FITC; Pacific Blue | BioLegend | A20 |
| CD45.2 | Surface marker | APC-eFluor 780 PE | Invitrogen | 104 |
Koleci et al., 2024

## Slide 18
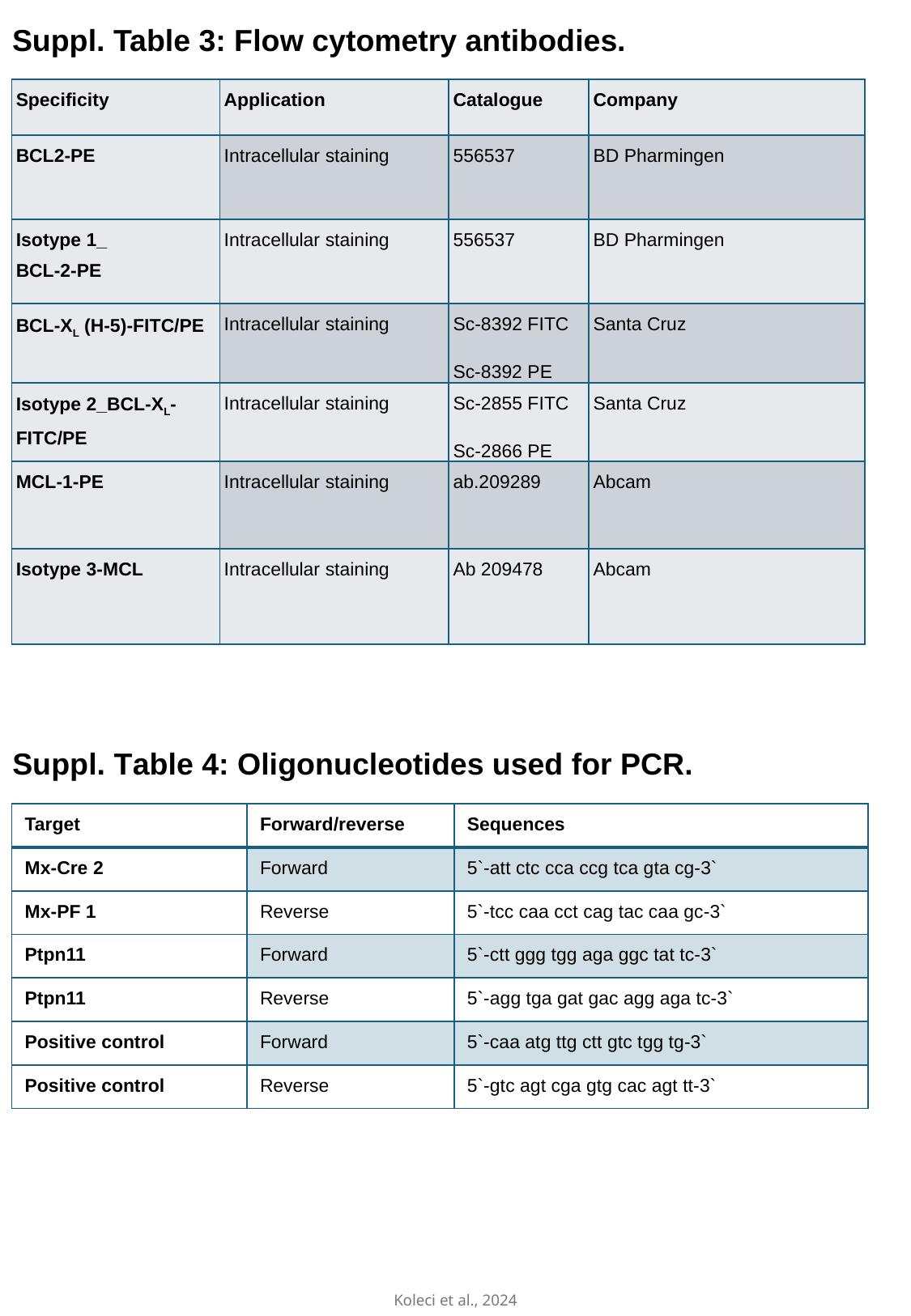

# Suppl. Table 3: Flow cytometry antibodies.
| Specificity | Application | Catalogue | Company |
| --- | --- | --- | --- |
| BCL2-PE | Intracellular staining | 556537 | BD Pharmingen |
| Isotype 1\_ BCL-2-PE | Intracellular staining | 556537 | BD Pharmingen |
| BCL-XL (H-5)-FITC/PE | Intracellular staining | Sc-8392 FITC Sc-8392 PE | Santa Cruz |
| Isotype 2\_BCL-XL-FITC/PE | Intracellular staining | Sc-2855 FITC Sc-2866 PE | Santa Cruz |
| MCL-1-PE | Intracellular staining | ab.209289 | Abcam |
| Isotype 3-MCL | Intracellular staining | Ab 209478 | Abcam |
Suppl. Table 4: Oligonucleotides used for PCR.
| Target | Forward/reverse | Sequences |
| --- | --- | --- |
| Mx-Cre 2 | Forward | 5`-att ctc cca ccg tca gta cg-3` |
| Mx-PF 1 | Reverse | 5`-tcc caa cct cag tac caa gc-3` |
| Ptpn11 | Forward | 5`-ctt ggg tgg aga ggc tat tc-3` |
| Ptpn11 | Reverse | 5`-agg tga gat gac agg aga tc-3` |
| Positive control | Forward | 5`-caa atg ttg ctt gtc tgg tg-3` |
| Positive control | Reverse | 5`-gtc agt cga gtg cac agt tt-3` |
Koleci et al., 2024
