# Supplementary material for: Oncogenic and microenvironmental signals drive cell type specific apoptosis resistance in juvenile myelomonocytic leukemia
Source: Cell Death Dis. 2025 Mar 8;16(1):165. doi: 10.1038/s41419-025-07479-2 (PMC11890777; doi:10.1038/s41419-025-07479-2)
Supplement: Supplementary file 2 — WB_original pictures. [file 41419_2025_7479_MOESM2_ESM.pdf]

WB
original pictures

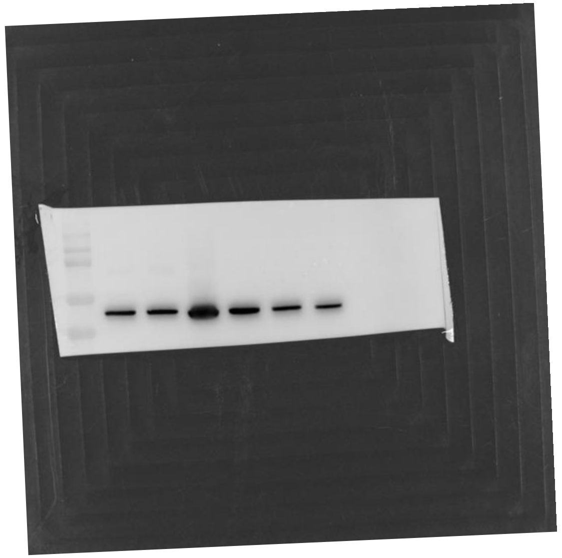

β-actin

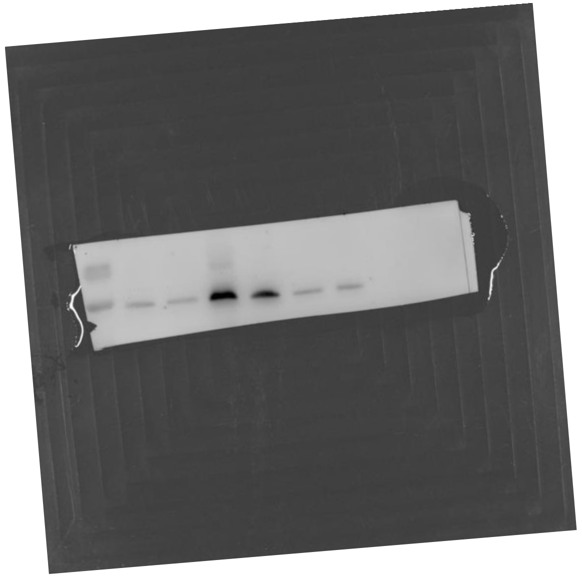

BAX

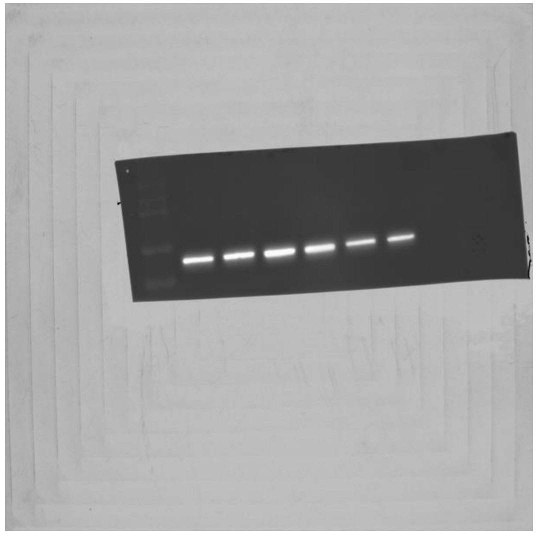

ß-actin

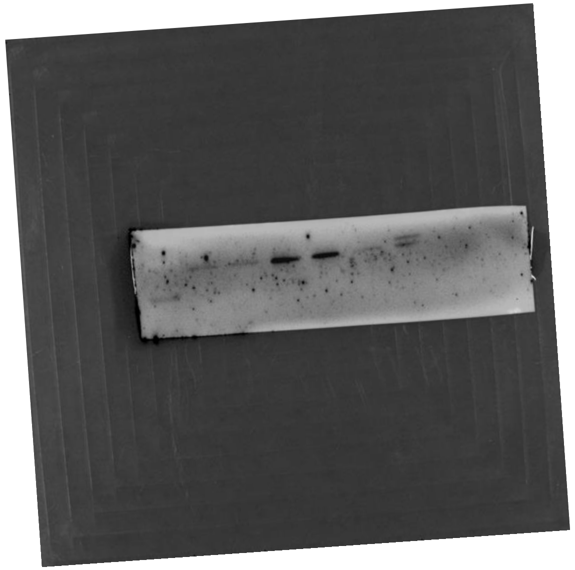

BAK

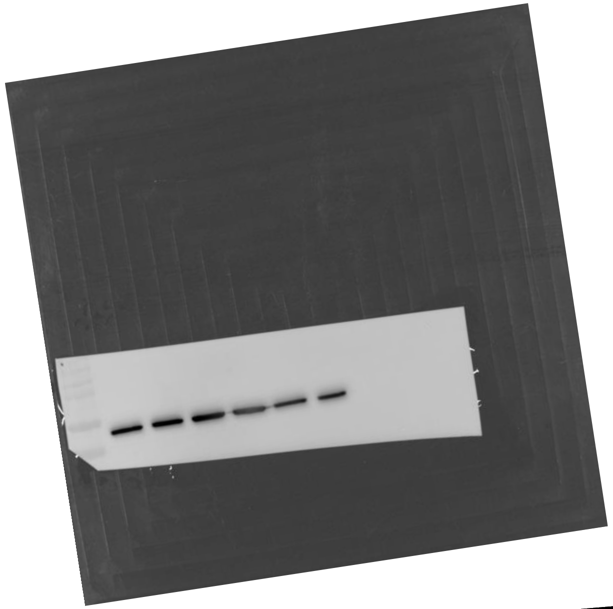

β-actin

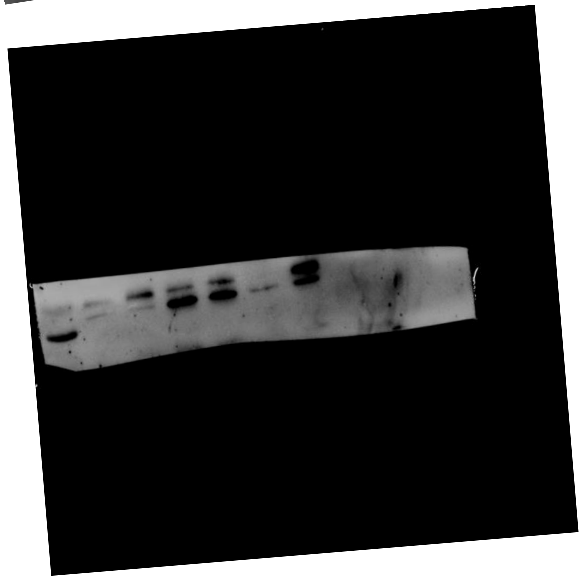

BID

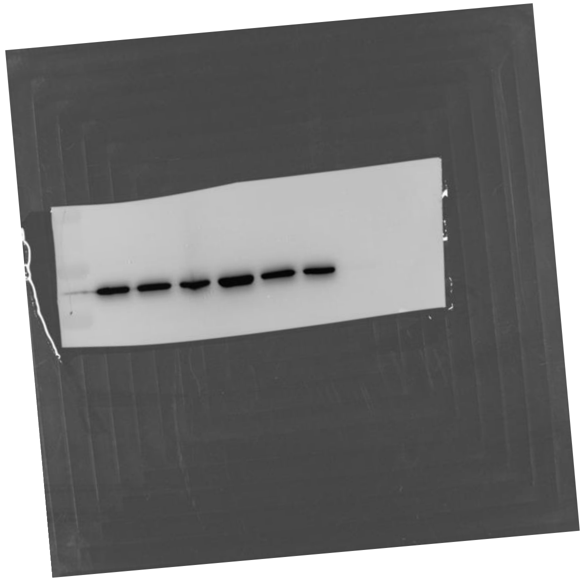

β-actin

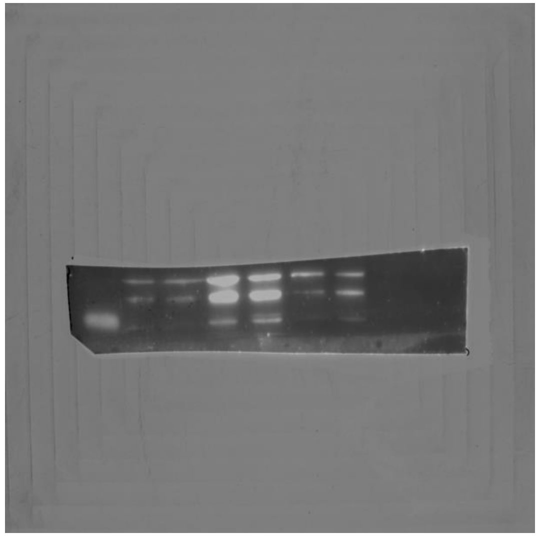

BMF

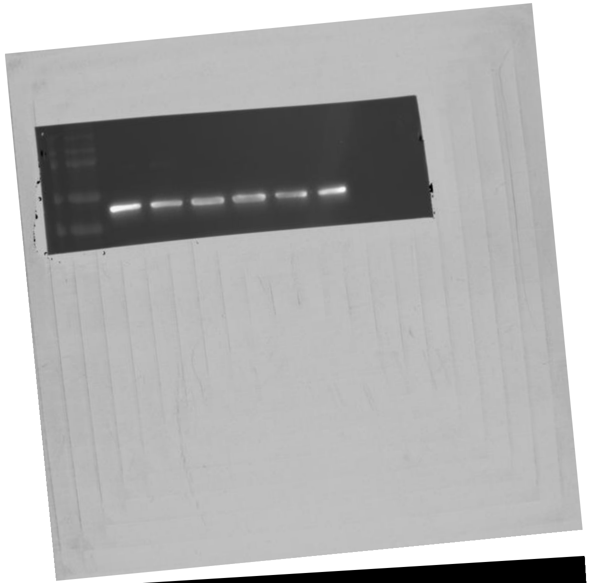

ß-actin

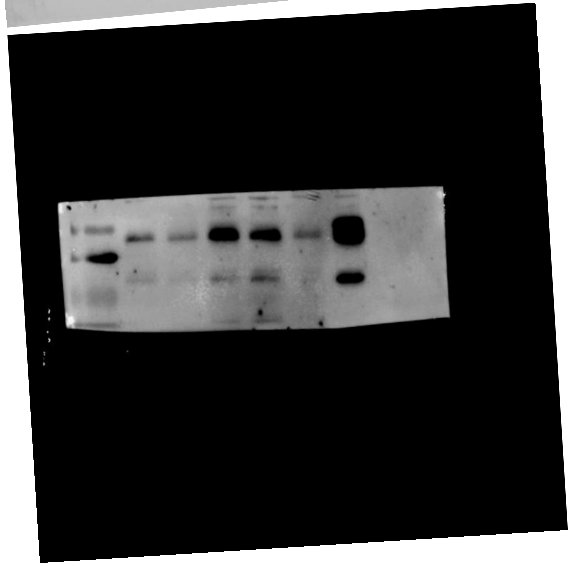

BIM

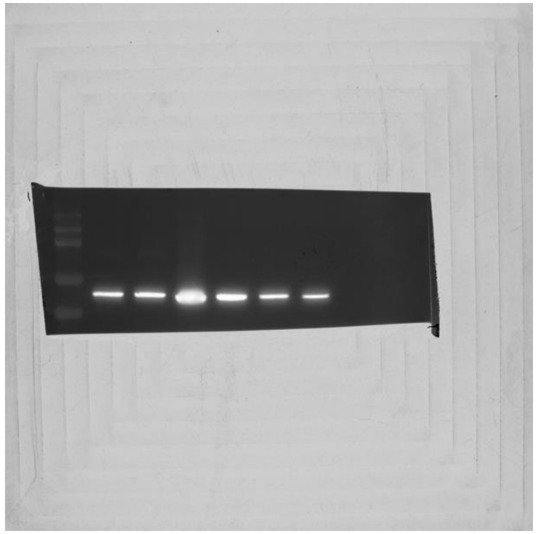

ß-actin

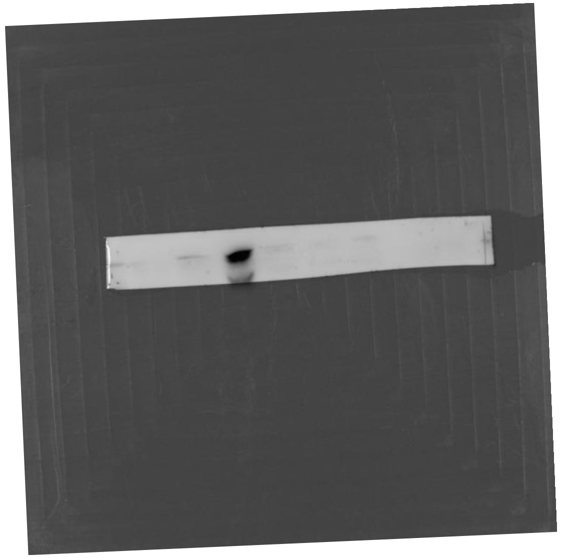

NOXA
